# A helical fulcrum in eIF2B coordinates allosteric regulation of stress signaling

**DOI:** 10.1038/s41589-023-01453-9

**Published:** 2023-11-09

**Authors:** Rosalie E. Lawrence, Sophie R. Shoemaker, Aniliese Deal, Smriti Sangwan, Aditya A. Anand, Lan Wang, Susan Marqusee, Peter Walter

**Affiliations:** 1 Department of Biochemistry and Biophysics, University of California, San Francisco, San Francisco, CA USA; 2 Howard Hughes Medical Institute, University of California, San Francisco, San Francisco, CA USA; 3 Department of Molecular and Cell Biology, University of California, Berkeley, Berkeley, CA USA; 4 Department of Chemistry, University of California, Berkeley, Berkeley, CA USA; 5 Chan Zuckerberg Biohub, San Francisco, CA USA; 6 California Institute for Quantitative Biosciences, University of California, Berkeley, Berkeley, CA USA; 7 Present Address: Altos Laboratories, Bay Area Institute of Science, Redwood City, CA USA; 8 Present Address: Genentech, Inc., South San Francisco, CA USA; 9 Present Address: The Hong Kong University of Science and Technology, Hong Kong, Hong Kong

**Keywords:** Enzyme mechanisms, Cell signalling, Structural biology, Mass spectrometry, Cell death

## Abstract

The integrated stress response (ISR) enables cells to survive a variety of acute stresses, but chronic activation of the ISR underlies age-related diseases. ISR signaling downregulates translation and activates expression of stress-responsive factors that promote return to homeostasis and is initiated by inhibition of the decameric guanine nucleotide exchange factor eIF2B. Conformational and assembly transitions regulate eIF2B activity, but the allosteric mechanisms controlling these dynamic transitions and mediating the therapeutic effects of the small-molecule ISR inhibitor ISRIB are unknown. Using hydrogen–deuterium exchange–mass spectrometry and cryo-electron microscopy, we identified a central α-helix whose orientation allosterically coordinates eIF2B conformation and assembly. Biochemical and cellular signaling assays show that this ‘switch-helix’ controls eIF2B activity and signaling. In sum, the switch-helix acts as a fulcrum of eIF2B conformational regulation and is a highly conserved actuator of ISR signal transduction. This work uncovers a conserved allosteric mechanism and unlocks new therapeutic possibilities for ISR-linked diseases.

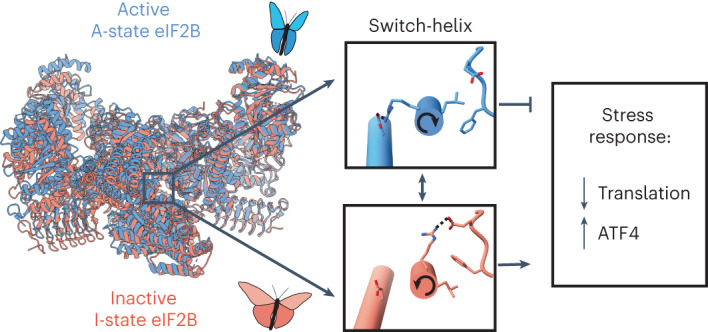

## Abstract (web summary)

Hydrogen–deuterium exchange–mass spectrometry and cryo-EM analysis revealed an allosteric mechanism involving the conformation of a single α-helix that controls the global conformation and activity of eIF2B, the core molecular machine of the integrated stress response.

## Main

The integrated stress response (ISR) is a conserved signaling network that promotes cellular fitness in response to biological stress by reprogramming translation and metabolism^1,2^. Acute activation of the ISR downtunes general protein synthesis while selectively activating the expression of stress-responsive factors, including chaperones, redox balancers and amino acid importers^2^. If a return to homeostasis is not achieved, ISR signaling can activate apoptosis^3^. Although acute activation of the ISR is adaptive, a chronic ISR signaling state is associated with a wide range of age-related and neurodegenerative pathologies, including Alzheimer’s disease, brain injury-induced dementia, cancer and Down syndrome^4–10^. ISR activation can be attenuated by ISRIB, a potent small drug-like molecule with dramatic effects on cognitive function in a wide variety of pathological states, but the allosteric mechanism by which ISRIB controls its target, eIF2B, is unknown^5–7^.

**Fig. 1:**
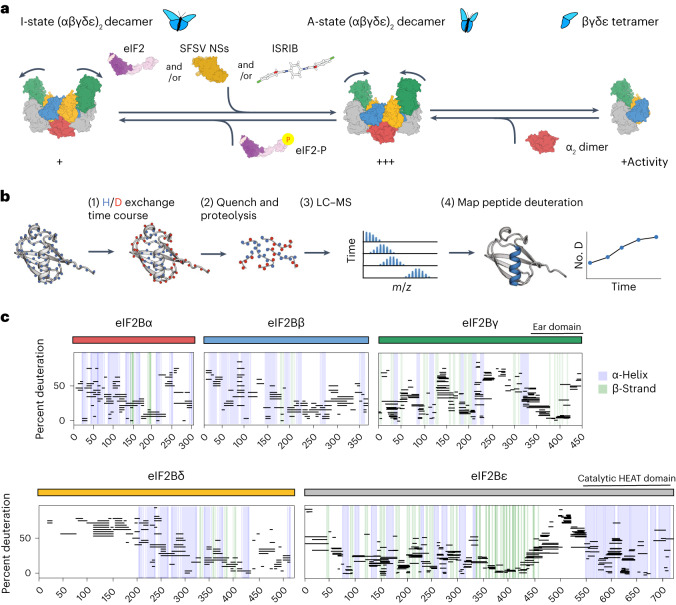
HDX–MS probes eIF2B structure. **a**, eIF2B is regulated by conformation and assembly state. Conversion to the less active I-state conformation (left) is driven by eIF2-P binding. Stabilization of the active A-state conformation (middle) is driven by eIF2, NSs or ISRIB binding; SFSV, sandfly fever Sicilian Assembly from less active tetramers (right) into more active decamers (middle) is driven by availability of the eIF2Bα_2_ dimer. **b**, Schematic of an HDX–MS experiment. Protein is incubated in deuterated solvent, and amide hydrogens (H) are able to exchange with deuterium (D) until defined time points (1). Exchange is quenched via pH and temperature drop, and protein is protease digested (2). Average peptide deuteration is detected via LC–MS (3). Peptide deuteration uptake is plotted over time and interpreted in the context of structural information (4). **c**, Percent deuteration after 100 s of deuterium labeling for every peptide in one apo eIF2B dataset. Solid-colored bars indicate each eIF2B subunit, corresponding to the color scheme in **a**. Each horizontal line represents an individual peptide spanning the residues indicated on the *x* axis, with percent deuteration (not correcting for back exchange) indicated on the *y* axis. α-Helices are indicated by blue vertical lines, and β-strands are indicated by green vertical lines, derived from the apo eIF2B structure PDB 7L70. Regions not resolved in the apo structure for which secondary structural information exists include the eIF2Bγ C-terminal ‘ear domain’ (secondary structure derived from PDB 7D44) and the eIF2Bε C-terminal HEAT domain (secondary structure derived from PDB 6O81). Shown are peptides from one representative experiment; all HDX experiments were replicated at least three independent times.

The ISR’s complex circuitry drives downstream signaling by controlling the rate of translation initiation complex assembly^11^. The rate-determining step in this pathway is set by eIF2B, a heterodecameric guanine nucleotide exchange enzyme whose activity is determined by its conformational state and its assembly from subcomplexes (Fig. 1a)^12–14^. An important goal is to uncover the molecular mechanisms that enable these dynamic transitions. For instance, how does binding of modulators communicate allosterically across a large multisubunit complex to regulate signaling? Defining molecular mechanisms of eIF2B control will illuminate basic principles of allosteric regulation of large signaling complexes and will open new avenues for therapeutic development.

eIF2B promotes GTP loading of the trimeric GTPase eIF2 (composed of α-, β- and γ-subunits)^15^, enabling eIF2 to assemble with methionyl-initiator tRNA to form the rate-limiting translation initiation ternary complex (TC)^16^. When TC levels are low, translation of most mRNAs is inhibited. A mechanism involving 5′-untranslated region regulation promotes stress-adaptive gene expression programs via selective translation of specific mRNAs, including the mRNA encoding the transcription factor ATF4 (refs. ^11,15,17^). We define the ISR as the events triggered in cells that contain limiting TC.

eIF2B is a central hub of ISR signaling^18,19^. Stress is sensed by the four distinct kinases PERK, HRI, GCN2 and PKR, which all phosphorylate eIF2α S51 in response to misfolded proteins, redox and mitochondrial stress, amino acid deficiency and viral infection, respectively^1^. Phosphorylation activates the ISR by converting eIF2 from a substrate of eIF2B (‘eIF2 substrate’) into its inhibitor (‘eIF2-P inhibitor’), impeding eIF2 GTP loading and hence TC assembly^20^. Therefore, eIF2B is a tunable ISR regulator whose activity is inversely proportional to ATF4 induction^18,19^.

**Extended Data Fig. 1:**
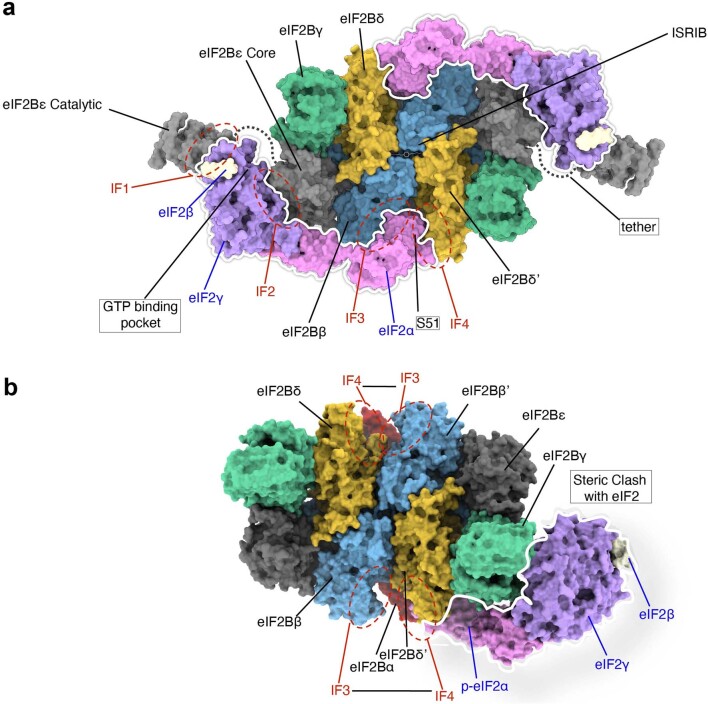
eIF2B enzyme engages eIF2 substrate and p-eIF2 inhibitor via distinct compound interfaces. **(a)** A surface representation of a model of two eIF2 trimers and ISRIB bound to an eIF2B(αβγδε)_2_ decamer is shown. Individual subunits of eIF2 and eIF2B are indicated. The eIF2 trimers are outlined in white and the locations of interfaces IF1-4 are indicated, as are the positions of eIF2 S51, the GTP binding pocket (empty in the structure), and ISRIB (shown in stick presentation). Note that in this productive, A-State complex, eIF2α binds between the eIF2Bβ and eIF2Bδ subunits, representing IF3 and IF4. The eIF2Bα_2_ dimer is hidden in this orientation. eIF2Bε contains two domains including the eIF2Bε catalytic HEAT domain that is linked by a flexible tether which was not resolved in the structure. (**b**) A surface representation of a model of one p-eIF2 trimer bound to eIF2B (PDB 6K72), representing the non-productive I-State complex. P-eIF2α binds between eIF2Bα and eIF2Bδ and prompts a widening of the pocket defined by substrate-binding interfaces IF3 and IF4, between the eIF2Bβ and eIF2Bδ subunits (indicated by black arrows). Steric conflict prevents eIF2 substrate from binding to the same half of the eIF2B complex as p-eIF2 inhibitor; low-affinity binding of one eIF2 molecule on the other half of the complex to an altered IF3 interface remains possible.

eIF2B is a twofold symmetric heterodecamer composed of two copies each of subunits α, β, γ, δ and ε (refs. ^21–23^). eIF2B activity is controlled by two distinct structural changes: conformation and assembly state^13,14^. The eIF2-P inhibitor prompts conformational change in eIF2B by binding either of two (symmetrically identical) pockets between the eIF2Bα and eIF2Bδ subunits. Binding induces a complex-wide rocking motion in which the two symmetric eIF2B halves rotate away from each other, akin to the flapping of a butterfly’s wings (Fig. 1a). In turn, this widens symmetric pockets where the eIF2 substrates bind (located between the eIF2Bβ and eIF2Bδ subunits), reducing substrate engagement and eIF2B activity (Extended Data Fig. 1). We refer to the inhibited, non-productive ‘wings-down’ state as the ‘I-state’ and the active, productive ‘wings up’ state as the ‘A-state’ (Fig. 1a)^13,14^. In the absence of binding partners, cryo-electron microscopy (cryo-EM) data suggest that eIF2B primarily samples the A-state^14^. The A-state can be further stabilized by binding to eIF2 substrate, ISRIB and its analogs or the viral ISR-inhibiting protein NSs (refs. ^24,25^). The two states are negatively coupled; each state has an alternatively accessible binding site, such that the productive A-state has a properly formed eIF2-binding pocket, and the non-productive I-state has a properly formed eIF2-P-binding pocket^13,14^.

eIF2B activity can also be regulated by assembly state in vitro. The decameric complex can disassemble into two stable tetramers composed of subunits β, γ, δ and ε (herein referred to as ‘eIF2B tetramer’ or ‘eIF2Bβγδε’) and one dimer composed of two α-subunits (herein referred to as ‘eIF2Bα_2_’ or ‘α_2_ dimer’; Fig. 1a)^18,19,21^. eIF2B tetramers have reduced activity relative to fully assembled eIF2B decamers (later referred to as ‘eIF2B(αβγδε)_2_’ or ‘eIF2B decamers’). Thus, it has been proposed that cells could tune ISR activity by regulating eIF2Bα_2_ availability, controlling the amount of fully active eIF2B decamers, although the relative population of eIF2B tetramers with respect to eIF2B decamers appears to be low in many cases^13,14^. The small-molecule ISRIB inhibits the ISR by binding across the tetramer–tetramer interface, promoting both eIF2B decameric assembly and stabilization of the active A-state^21,22,26,27^. Notably, tetrameric eIF2B has an activity that approximates that of the I-state eIF2B decamer because, as previously posited, the I-state rocking motion removes one of four eIF2 interaction interfaces (interface 4 in Extended Data Fig. 1)^14^.

Presently, it is unknown how binding of activators or inhibitors at distant sites coordinates allosteric remodeling across the multiple subunits of eIF2B, ultimately controlling its activity and ISR signaling. An important goal is to uncover the mechanisms enabling these dynamic transitions. Here, we use hydrogen–deuterium exchange–mass spectrometry (HDX–MS), cryo-EM and biochemistry to map the allosteric mechanism controlling eIF2B activity. We propose a conserved mechanism that regulates both eIF2B conformational and assembly transitions centered around our discovery of the eIF2B ‘switch-helix’.

## Results

### Hydrogen–deuterium exchange probes eIF2B conformations

To define molecular mechanisms of the regulatory transitions of eIF2B, we used HDX–MS to obtain a comprehensive profile of eIF2B’s structural flexibility. HDX–MS monitors the exchange of protein backbone amide hydrogens for deuterium atoms from a deuterated buffer at the resolution of small peptides^28^. An amide’s rate of exchange is directly related to its solvent accessibility and local stability, reporting on changes in structure and local stability^29–31^. HDX–MS experiments are performed by exposing protein to deuterated solvent (Fig. 1b, step 1), quenching the deuteration reaction at various time points (via low pH and low temperature), proteolyzing the sample under quenched conditions (step 2), followed by in-line liquid chromatography–mass spectrometry (LC–MS; step 3) to detect the number of deuterons per peptide at each time point. The changes in mass due to uptake of deuterons can then be mapped onto the structure, revealing time-resolved structure and conformational stability (step 4).

**Extended Data Fig. 2:**
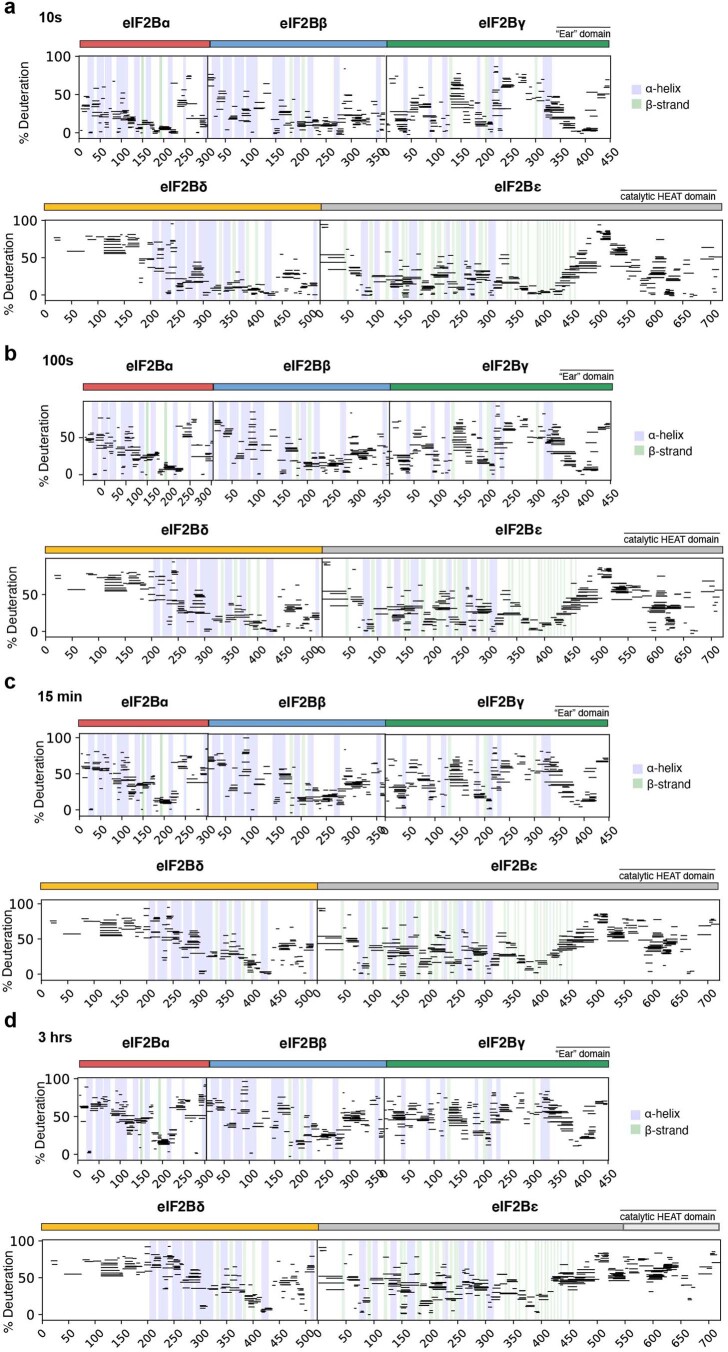
eIF2B deuteration over time. Percent deuteration after (**a**) 10 seconds (**b**) 100 seconds (**c**) 15 minutes (**d**) 3 hours of deuterium exchange for every peptide in one apo eIF2B dataset. Solid colored bars indicate each eIF2B subunit, corresponding to color scheme in Fig. 1. Each horizontal line represents an individual peptide spanning the residues indicated on the x axis, with percent deuteration (not correcting for back exchange) indicated on the y axis. α-helices are indicated in blue vertical lines, and β-strands are indicated in green vertical lines, derived from apo eIF2B structure PDB 7L70. Shown are peptides from one representative experiment; all HX experiments were replicated at least 3 independent times.

**Extended Data Fig. 3:**
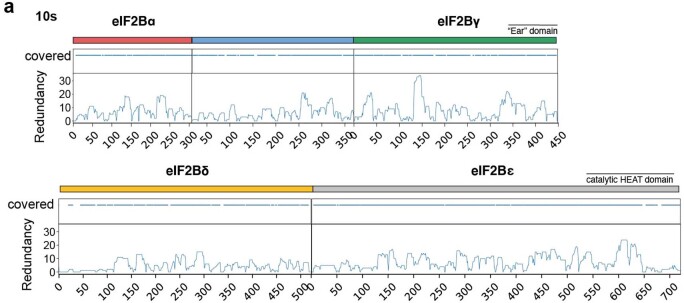
Coverage and redundancy results from HDX-MS experiments. (**a**) coverage and redundancy at each eIF2B residue for peptides included in all conditions of the comparative apo eIF2B vs NSs-bound eIF2B vs eIF2-P-bound eIF2B experiment. Solid colored bars indicate each eIF2B subunit, corresponding to color scheme in Fig. 1. Shown are peptides from one representative experiment; all HX experiments were replicated at least 3 independent times.

After extensive optimization of protease digestion and quench conditions, we obtained excellent sequence coverage (92%) of the large eIF2B decamer (2,369 unique amino acids; Supplementary Table 1). This enabled monitoring of nearly the entire sequence space, including conformationally flexible regions not modeled in existing cryo-EM structures (Fig. 1c and Extended Data Figs. 2 and 3)^13,18,21,22,24,25^. As expected, we observed broad agreement between regions of HDX–MS protection and experimental secondary structure assignment based on the cryo-EM structure of apo eIF2B (Fig. 1c)^14^.

### Hydrogen–deuterium exchange identifies one remodeled helix from two transitions

**Fig. 2:**
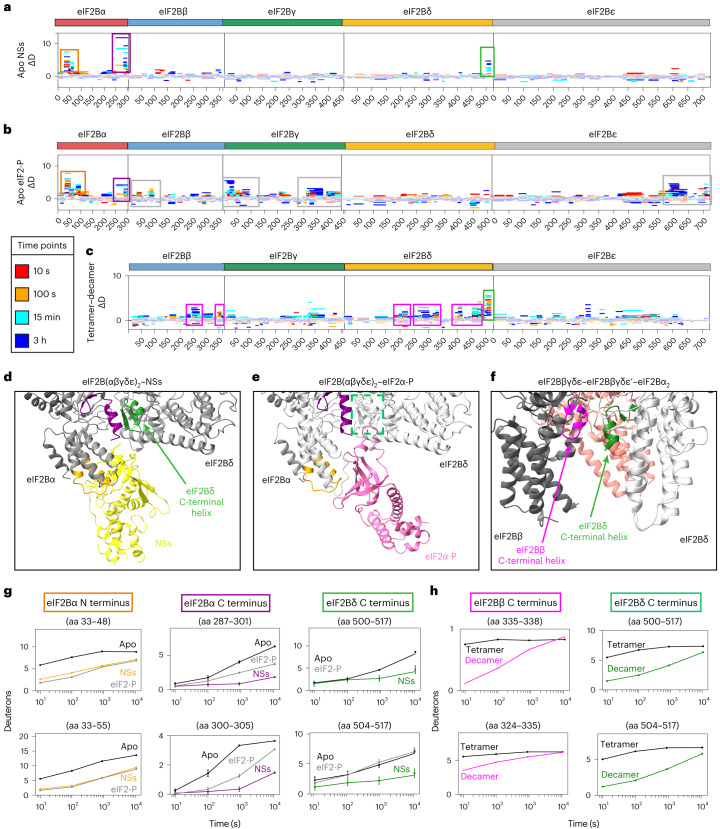
HDX–MS analysis of eIF2B conformation and assembly states identifies remodeling of the same helix. **a**–**c**, Representative deuteration difference maps for apo eIF2B versus NSs-bound eIF2B (**a**), apo eIF2B versus eIF2-P-bound eIF2B (**b**) and eIF2Bβγδε tetramer versus eIF2B(αβγδε)_2_ decamer (**c**). On each graph, time points are overlayed and color coded. The 10-s deuteron difference is mapped in red, the 100-s difference is mapped in orange, the 15-min difference is mapped in cyan, and the 3-h difference is mapped in dark blue. Positive values represent peptides with more protection (less deuteration) in the second listed state than in the first listed state. Significant protection is defined as multiple peptides with a change in number of deuterons (∆D) of greater than 0.5; peptides without significant protection are contained within the dimmed threshold. Regions of NSs and eIF2-P protection located at the eIF2Bα effector binding site are shown in orange and purple boxes. The eIF2Bδ C-terminal switch-helix is shown in green boxes. eIF2-P-dependent protection of eIF2Bβ, eIF2Bγ and eIF2Bε is indicated by gray boxes. eIF2B regions of protection at the decamerization interface are indicated by pink boxes. Shown are peptides from one representative experiment; all HDX experiments were replicated at least three independent times. **d**–**f**, Structural maps of the NSs binding pocket (**d**), eIF2α-P binding pocket (**e**) and tetramer–tetramer interface (**f**) of the eIF2B(αβγδε)_2_ decamer. Regions of interest are color coded corresponding to protected regions shown in deuteration difference plots (see **a**–**c**) and representative peptide uptake plots (see **g** and **h**). **g**,**h**, Peptide uptake plots showing the average number of exchanged deuterons per condition over time for representative peptides (representing an average of three independent experiments; error bars represent s.e.m.; not back-exchanged corrected); aa, amino acids.

**Extended Data Fig. 4:**
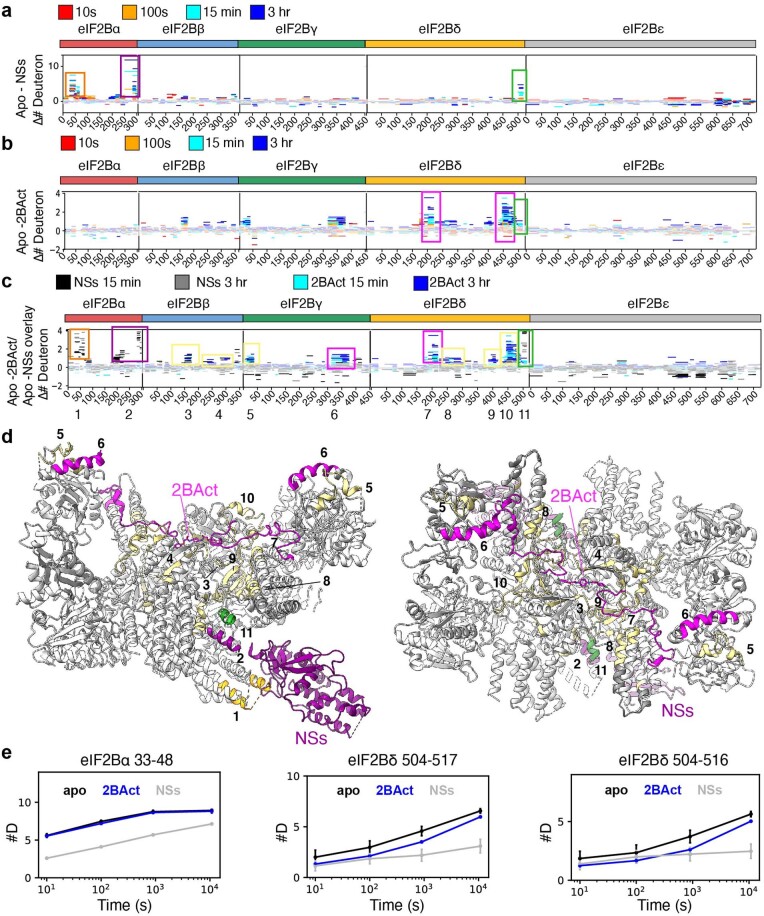
Comparison of eIF2B HDX-MS protection by NSs and 2BAct. **a**,**b**, Representative percent deuteration difference map with overlaid time points for (**a**) apo versus NSs-bound eIF2B(αβγδε)_2_ decamers and (**b**) apo versus 2BAct-bound eIF2B(αβγδε)_2_ decamers. Positive values represent peptides that exchanged less deuterons in the 2BAct—bound state. Solid colored bars indicate each eIF2B subunit, corresponding to color scheme in Fig. 1. On each graph, timepoints are overlayed and color-coded: 10s deuteron difference mapped in red, 100s difference mapped in orange, 15 min difference mapped in cyan, 3 hr difference mapped in dark blue. Positive values represent peptides with more protection (less deuteration) in the second listed state relative to the first listed state. Significant protection is defined as multiple peptides with ∆#D greater than 0.5 deuterons; peptides without significant protection are contained within the dimmed threshold. Regions of NSs protection located at eIF2Bα effector binding site are shown in orange and purple boxes. The 2BAct effector binding site is indicated by pink boxes. The eIF2Bδ C-terminal ‘Switch-Helix’ is indicated by green boxes. (**c**) apo versus 2BAct-bound eIF2B(αβγδε)_2_ decamers and apo versus NSs-bound eIF2B(αβγδε)_2_ decamers difference maps plotted onto the same ∆#D axes. Timepoints are overlaid and color-coded; NSs 15 min is mapped in black, NSs 3 hr is grey, 2BAct 15 min is cyan, and 2BAct 3 hr is dark blue. Regions of NSs protection located at eIF2Bα effector binding site are shown in orange and purple numbered boxes. The eIF2Bδ C-terminal ‘Switch-Helix’ is indicated by green numbered box. Regions specifically protected by 2BAct indicated with pink numbered boxes; regions protected by both 2BAct and NSs are indicated with yellow numbered boxes. (**d**) Numbered regions from (**c**) are mapped onto eIF2B-NSs structure (PDB 7RLO) with 2BAct binding site shown. (**e**) Representative peptide uptake plots comparing 2BAct-bound (blue) vs NSs-bound (grey) vs apo eIF2B (black). The total number of exchanged deuterons per condition are plotted over time (n = three independent experiments) ± SD not corrected for back exchange.

To uncover underlying mechanisms that control the eIF2B A-state → I-state conformational change and/or its tetramer → decamer assembly, we performed comparative HDX–MS experiments. To this end, we biased the population of eIF2B decamers toward the A-state with the addition of the viral ISR inhibitor NSs or the small-molecule ISRIB analog 2BAct and performed HDX–MS to compare the time-dependent deuterium uptake between the apo state and the A-state (Fig. 2a and Extended Data Fig. 4). Similarly, we biased eIF2B decamers toward the I-state by the addition of eIF2-P and again compared deuterium uptake to the apo state (Fig. 2b). A summary of all HDX–MS data is shown in Fig. 2. We defined a region as showing increased protection if multiple peptides showed less (by at least 0.5 deuterons) deuterium uptake in the defined A-state or I-state than in the apo state. Differential deuteration of peptides across all regions of eIF2B are plotted (Fig. 2a–c), peptides showing increased protection are mapped onto the corresponding cryo-EM structure (Fig. 2d–f), and representative uptake profiles show individual peptide deuteration over time (Fig. 2g,h).

First, we noted protection of peptides in regions where modulators and eIF2B physically interact. Comparing apo eIF2B to the NSs-stabilized A-state eIF2B, we observed increased protection for peptides in eIF2Bα that map to the known NSs binding interface (Fig. 2a,d,g and Extended Data Fig. 4a,c; orange and purple indicate regions of NSs binding to eIF2Bα). Similarly, we observed protection of known eIF2-P binding interfaces when comparing apo eIF2B to the eIF2-P-induced eIF2B I-state. The phosphorylated α-subunit of eIF2-P binds in the same pocket targeted by NSs but forms additional contacts with the eIF2Bδ subunit to stabilize the I-state rather than the A-state (Fig. 2b,e,g; orange and purple indicate regions of eIF2-P binding to eIF2Bα). The extended eIF2-P trimer also forms contacts and regions of increased protection on the eIF2Bγ subunit, which are consistent with structural models (Fig. 2b; gray boxes indicate regions of eIF2-P binding to eIF2B). We note that eIF2-P increased protection in eIF2Bε peptides, specifically toward its C-terminal HEAT domain. This may represent a contact not resolved in current cryo-EM structures, possibly via the eIF2β subunit (Fig. 2b, right-most gray box). We also observed protection in peptides at the 2BAct binding site (a groove between eIF2Bβ and eIF2Bδ′ on the opposite face of eIF2B henceforth referred to as the ‘ISRIB pocket’) for the 2BAct-stabilized eIF2B A-state but not for peptides at the NSs binding interface (Extended Data Fig. 4b; pink boxes indicate regions of 2BAct binding to eIF2B).

Notably, we also observed regions of protection not corresponding to modulators’ direct binding interfaces, likely resulting from allosteric modulation. One prominent unexpected protection in the NSs-bound A-state mapped to the most C-terminal eIF2Bδ helix (Fig. 2a,d,g; indicated in green). Similarly, in the A-state stabilized by 2BAct, we saw the same effect on the C-terminal eIF2Bδ helix, albeit to a lower extent (Extended Data Fig. 4b–e). Importantly, this region does not show increased protection in the eIF2-P-bound I-state (Fig. 2b,e,g). These results are surprising because the C-terminal eIF2Bδ helix does not make direct contact with NSs or 2BAct (Fig. 2d), suggesting that biasing eIF2B into the A-state causes protection allosterically rather than by direct ligand contact. A network of regions within eIF2Bβ, eIF2Bδ and eIF2Bγ connecting the ISRIB pocket, inhibitor binding site and eIF2Bγ ‘ear’ domain also showed increased protection in both the NSs-bound and 2BAct-bound datasets, which may likewise have a role in allosteric communication across eIF2B (indicated in yellow; Extended Data Fig. 4b,c). These regions are particularly apparent in the 2BAct dataset, which shows a relatively lower degree of protection for the C-terminal eIF2Bδ helix.

The C-terminal eIF2Bδ helix is located at the decamerization interface, at which the C-terminal helices of eIF2Bα, eIF2Bβ and eIF2Bδ cluster (Fig. 2h; the eIF2Bδ C-terminal helix is shown in green, and the eIF2Bβ C-terminal helix is shown in pink). We next asked what local structural rearrangements mediate assembly of the eIF2B decamer by monitoring deuteration of eIF2B tetramers alone versus those mixed with eIF2Bα_2_, assembling the decamer. Remarkably, peptides in the same eIF2Bδ C-terminal helix region emerged as the most differentially protected regions in the decamer, including relative to other peptides located at the decamerization interface (Fig. 2c,f,h).

Collectively, these data indicate that the eIF2Bδ C-terminal helix undergoes conformational changes in both the A-state → I-state and tetramer → decamer eIF2B transitions. In the tetramer, the amide protons in this helix exchange rapidly, suggesting that it is predominantly unfolded (Fig. 2h). After decamerization (to the apo-state decamer), these amide protons show increased protection on the timescale of the HDX experiment. Relative to the apo-state decamer, we see more rapid protection of the eIF2Bδ C-terminal helix in the A-state decamer (Fig. 2g). These data indicate that this helix undergoes conformational and energetic changes in both assembly and A-state → I-state transitions, suggesting that it may play a crucial role in allosteric regulation of eIF2B activity. Therefore, we probed the ability of specific molecular interactions with the helix’s side chains to control eIF2B activity, conformation and assembly.

### The eIF2Bδ C-terminal helix is a conformational switch

**Fig. 3:**
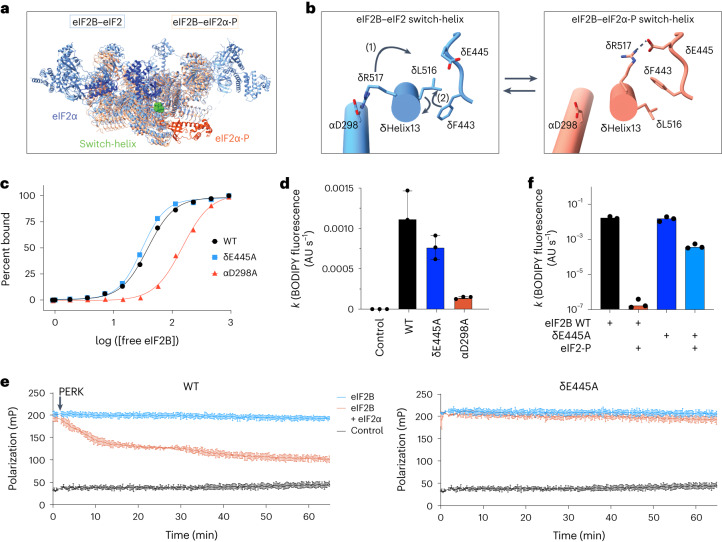
The eIF2Bδ C-terminal helix is a conformational switch. **a**, Overview of atomic models of eIF2-bound eIF2B (eIF2B in light blue and eIF2α in dark blue; PDB 6O81) and eIF2-P-bound eIF2B (eIF2B in salmon and eIF2α-P in dark orange; PDB 6O9Z) with the eIF2B C-terminal switch-helix indicated in green; [free eIF2B], concentration of free eIF2B. **b**, The eIF2Bδ C-terminal helix undergoes a conformational change mediated by remodeled side chain interactions in the A-state → I-state transition. In the A-state (left; PDB 6O81), δR517 forms a salt bridge (dotted lines) with αD298, and the δF443 rotamer is in the ‘down’ position. (1) In the I-state (right; PDB 6O9Z), δR517 forms a new salt bridge (dotted lines) with δE445, which coincides with (2) rotation of the eIF2Bδ C-terminal helix and adoption of the δF443 ‘up’ rotameric state. **c**, Binding assay for fluorescent FAM-ISRIB interaction with eIF2B(αβγδε)_2_ decamers with the indicated mutations using fluorescence polarization (calculated half-maximal effective concentration values (95% confidence interval): WT = 38 ± 2 nM; δE445A = 30 ± 2 nM; αD298A = 152 ± 18 nM). **d**, BODIPY-GDP nucleotide loading assay of eIF2B(αβγδε)_2_ decamers (final concentration of 5 nM) with the indicated point mutation. Shown are averages and s.e.m. for three experimental replicates; AU, arbitrary units. **e**, Kinetic fluorescence polarization dissociation assay for fluorescent FAM-ISRIB preincubated with eIF2B(αβγδε)_2_ decamers. At time zero, PERK kinase domain was spiked into the assay. Shown are averages and s.e.m. for three experimental replicates. **f**, BODIPY-GDP nucleotide unloading assay of eIF2B(αβγδε)_2_ decamers (final concentration of 5 nM) with or without point mutations and with or without the addition of 25 nM eIF2-P, as indicated. Shown are averages and s.e.m. of rate constants (*k*) derived from a single exponential fit for three experimental replicates. Source data

**Extended Data Fig. 5:**
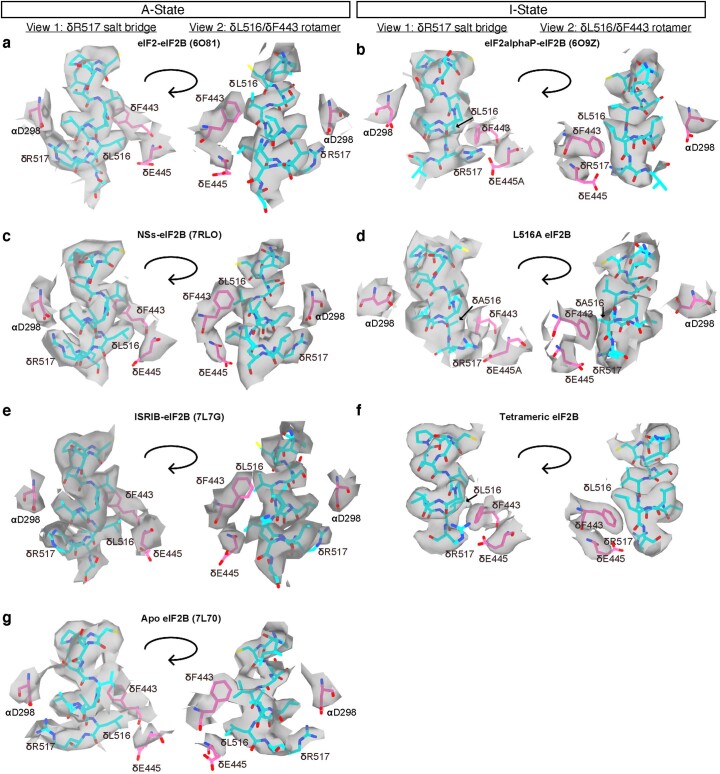
EM density surrounding eIF2Bδ Switch-Helix sidechains. Shown are EM density and atomic models of the eIF2Bδ Switch-Helix (cyan) and interacting sidechains (pink). Density zones were set to a radius of $2.5\, {\text{\AA }}$, and two representative views (left clearly displaying δR517 salt-bridge density and right clearly displaying δL516/δF443 rotamer density) are shown for each structure incuding: (**a**) eIF2-bound decameric A-state (PDB 6o81, EMD0649), (**b**) eIF2αP-bound decameric I-state (PDB 6o9z, EMD0664), (**c**) the NSs-bound decameric A-State (PDB 7RLO, EMD24235), (**d**) the L516A decameric I-State (PDB 8TQZ, EMD-41566), (**e**) the ISRIB-bound decameric A-State (PDB 7L7G, EMD7443), (**f**) the tetrameric state (PDB 8TQO, EMD 41510), (g) the decameric Apo eIF2B A-like state (PDB 7L70, EMD23209).

**Extended Data Fig. 6:**
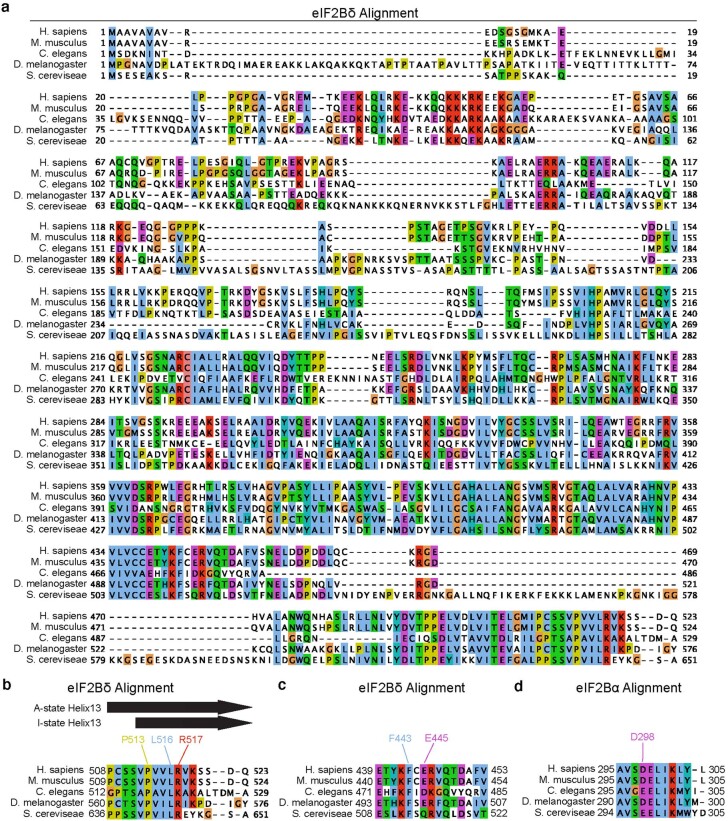
Sequence conservation of eIF2B Switch-Helix residues. (**a**) Multiple sequence alignment of eIF2Bδ from indicated organisms, with Clustal color-coding. (**b**) Multiple sequence alignment of eIF2B Switch-Helix from indicated organisms. Secondary structure is indicated above. (**c**) Multiple sequence alignment of eIF2Bδ residues that interact with the eIF2B Switch-Helix from indicated organisms. (**d**) Multiple sequence alignment of eIF2Bα residues that interact with the eIF2Bδ Switch-Helix from indicated organisms.

We first asked how this helix participates in the eIF2B A-state → I-state transition. Next, we investigated its role in the tetramer → decamer transition, discussed below. Comparison of A-state and I-state cryo-EM structures revealed remodeling of key side chain interactions (Fig. 3a, Extended Data Fig. 5 and Supplementary Video 1)^18,21^. In the A-state eIF2–eIF2B structure, eIF2Bδ R517 (δR517) forms a salt bridge (2.95-Å heavy atom distance) with eIF2Bα D298 (αD298; Protein Data Bank (PDB) 6O81). After transition to the I-state in response to eIF2-P binding to eIF2B, the entire helix, including δR517, rotates by 38°. The δR517–αD298 salt bridge breaks, and δR517 engages in an alternate salt bridge (2.65-Å heavy atom distance) with eIF2Bδ E445 (δE445; step 1 in Fig. 3b; PDB 6O9Z). Concomitantly, eIF2Bδ L516 (δL516) also rotates 38˚, and eIF2Bδ F443 (δF443) flips to an alternate rotamer position, avoiding steric clashes (step 2 in Fig. 3b). The eIF2Bδ C-terminal helix sequence is highly conserved (Extended Data Fig. 6). The side chain arrangement reported in eIF2–eIF2B is observed in all A-state structures (including NSs-bound (PDB 7RLO) and ISRIB-bound (PDB 7L7Z) structures; Extended Data Fig. 5), suggesting that these rearrangements reflect a general feature of the A-state → I-state transition.

We posit that helical rotation and rearrangement of the δR517 salt bridge and δF443 rotamer positions forms a concerted functional switch and hence refer to the eIF2Bδ C-terminal helix as the ‘switch-helix’. According to this view, triggering this switch is required for the A-state → I-state transition, with the switch-helix facilitating allosteric communication between the inhibitor binding site and other functional regions of eIF2B, such as the ISRIB and/or eIF2 substrate-binding pockets. To test this notion, we sought to bias the switch-helix toward a single conformation.

We first mutated αD298 to alanine (αD298A), aiming to promote the switch-helix I-state by preventing δR517 from engaging in the A-state salt bridge interaction. Indeed, as previously reported for the I-state, fluorescence polarization experiments revealed decreased binding of flourescent FAM-ISRIB to αD298A decamers (Fig. 3c)^14,22^, suggesting that the αD298A mutation induces the eIF2B I-state allosterically and remodels the ISRIB binding pocket. Consistent with this interpretation, αD298A also exhibited profoundly reduced nucleotide exchange activity (Fig. 3d). Using equivalent logic, we mutated δE445 to alanine (δE445A), thus removing the I-state salt bridge and biasing the switch-helix toward the A-state. Neither FAM-ISRIB binding nor nucleotide exchange activity were diminished (Fig. 3c,d), consistent with an A-state phenotype assumed by apo eIF2B (ref. ^14^).

Conversely, we predicted that biasing the switch-helix toward the A-state would render eIF2B more resistant to inhibition. To test this hypothesis, we assessed FAM-ISRIB binding in response to eIF2α-P inhibitor. As expected, whereas eIF2α-P decreased binding of FAM-ISRIB eIF2B wild-type (WT) decamers, δE445A decamers were insensitive to inhibition by eIF2α-P (Fig. 3e). Similarly, δE445A nucleotide exchange activity was less sensitive to inhibition by eIF2-P than eIF2B WT (Fig. 3f). These results support the view that the δR517–δE445 salt bridge plays an important role in stabilizing the I-state. In its absence, δE445A becomes biased toward the A-state, rendering eIF2-P inhibition less effective.

### Switch-helix δL516–δF443 rotamers control eIF2B conformation

The second conformational element of the switch-helix suggested from cryo-EM data concerns the interactions and rotameric disposition of δF443 and δL516. δF443 forms the ‘up’ rotamer position in the I-state and the ‘down’ rotamer position in the A-state, corresponding to a mirrored repositioning of δL516 (Fig. 3d). To explore the functional consequences of this rearrangement, we mutated δL516 and δF443 independently to alanine (δL516A and δF443A, respectively).

**Fig. 4:**
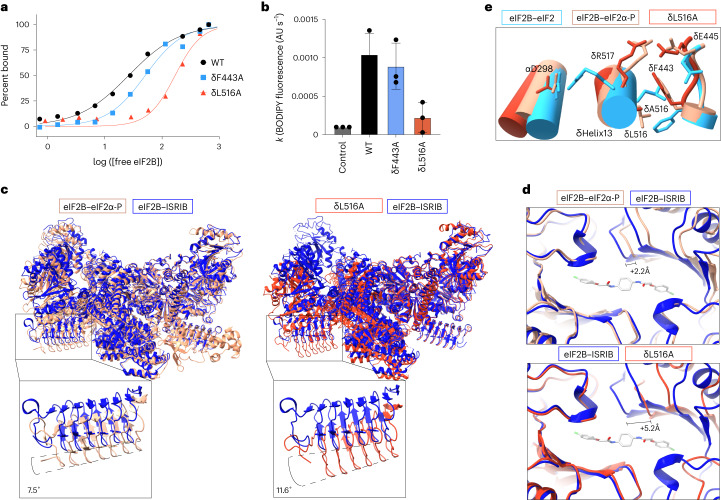
eIF2B switch-helix controls the A-state → I-state transition. **a**, Binding assay to assess fluorescent FAM-ISRIB interaction with eIF2B(αβγδε)_2_ decamers containing the indicated mutations using fluorescence polarization (calculated half-maximal effective concentration values (95% confidence interval): WT = 26 ± 3 nM; δF443A = 50 ± 8 nM; δL516A = 169 ± 34 nM). Shown is one of three experimental replicates. **b**, BODIPY-GDP nucleotide loading assay of eIF2B(αβγδε)_2_ decamers (final concentration of 5 nM) with the indicated point mutation. Shown are averages and s.e.m. of rate constants (*k*) derived from a single exponential fit for three experimental replicates. **c**, Atomic model of the ISRIB-bound A-state eIF2B model (PDB 7L7G; blue) overlaid on the eIF2α-P-bound I-state eIF2B model (PDB 6O9Z; peach) and δL516A decamer structure (PDB 8TQZ; dark orange). The inset shows a zoom-in view of the β-solenoid domain (residues 342–466) of eIF2Bε. The hinge movement between the two eIF2B halves was measured between the lines connecting eIF2Bε H352 and P439 in the indicated structures. **d**, Zoom-in view of the ISRIB binding pocket (PDB 7L7G), showing widening after eIF2α-P binding (peach) and further widening for the δL516A decamer (dark orange). The 2.2-Å and 5.2-Å pocket lengthening was measured between eIF2Bβ N162 and eIF2Bδ S178. **e**, Overlay of δL516A switch-helix side chains (dark orange) onto the eIF2-bound eIF2B A-state decamer (PDB 6O81; blue) and the eIF2α-P-bound eIF2B I-state (PDB 6O9Z; peach) atomic models. Source data

We first asked whether mutating these amino acids caused allosteric remodeling of the ISRIB pocket. Strikingly, we observed that the δL516A mutation resulted in a decrease in FAM-ISRIB binding affinity by almost an order of magnitude (eightfold), whereas the δF443A mutation reduced FAM-ISRIB binding by twofold (Fig. 4a). Similarly, the δL516A mutation strongly decreased nucleotide exchange activity, whereas the activity of δF443A was only slightly reduced (Fig. 4b). Thus in the absence of the steric clash driven by δL516, the δF443 rotamer and eIF2B as a whole default to the I-state orientation. If there is no δF443 rotamer clash (as for the δF443A mutant), the switch is broken, and eIF2B can assume either the A-state or I-state.

**Extended Data Fig. 7:**
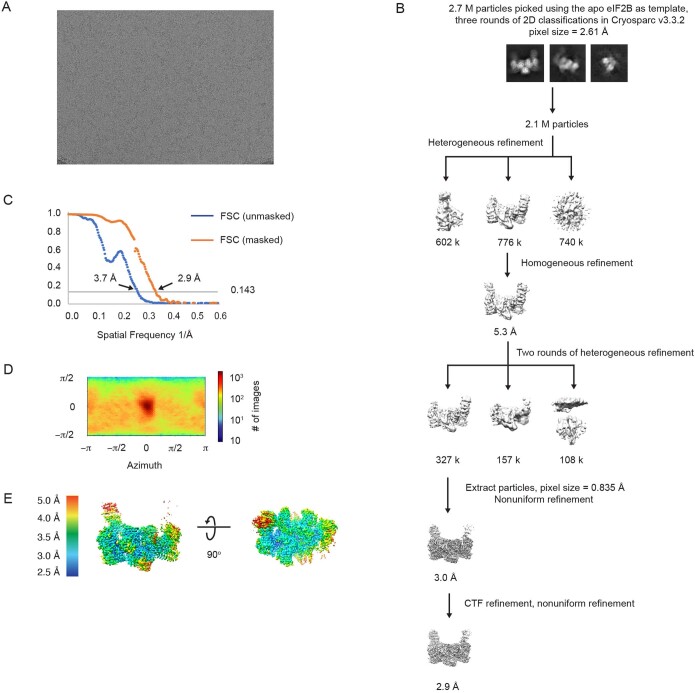
Cryo-EM data analysis of the eIF2B^δL516A^ structure. (**a**) Representative micrograph showing the quality of data used for the final reconstruction of the eIF2B^δL516A^ structure. A total of 4042 micrographs were acquired and analyzed. (**b**) Data processing scheme of the eIF2B^δL516A^ structure. (**c**) Fourier shell correlation (FSC) plots of the 3D reconstructions of eIF2B^δL516A^ unmasked (dark blue), masked (orange). (**d**) Orientation angle distribution of the eIF2B^δL516A^ reconstruction. (**e**) Local resolution map of the eIF2B^δL516A^ structure.

**Extended Data Fig. 8:**
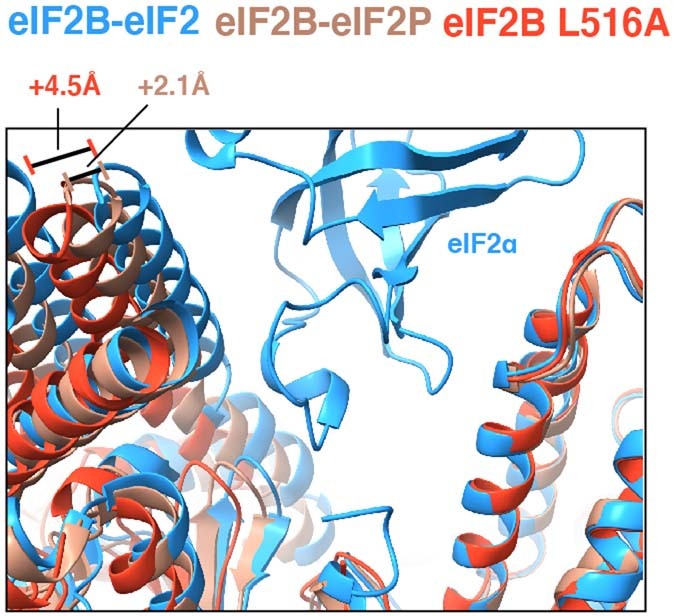
eIF2B δL516A mutation widens eIF2α binding pocket more than eIF2αP binding. Overlay of atomic models of eIF2B-eIF2 (blue, PDB 6o81), eIF2B-eIF2αP (light peach, PDB 6o9z), and eIF2B δL516A (orange, PDB 8TQZ) showing widening of eIF2α binding pocket by $2.1{\text{\AA }}$ for eIF2B-eIF2αP and 4.5 Å for eIF2B δL516A.

To further test this prediction, we determined a cryo-EM structure of eIF2B δL516A. After two-dimensional (2D) and three-dimensional (3D) classification, we generated a single consensus structure of the eIF2B δL516A decamer at a resolution of 2.9 Å (Supplementary Table 2 and Extended Data Fig. 7), with most side chain density clearly visible. We then built the atomic model of δL516A into this map. Consistent with our predictions, δL516A exhibited a ‘wings-down’ I-state-like conformation. The two tetramer subcomplexes underwent a rocking motion that changes the angle between them by 11.6° relative to the ISRIB-bound A-state (Fig. 4c). Indeed, δL516A adopted a more extreme I-state conformation than the previously reported doubley bound eIF2α-P eIF2B (tetrameric subcomplex rotation of 7.5°), the I-state-like eIF2Bβ-H160D mutant (tetrameric subcomplex rotation of 3.5°) or the singly bound eIF2α-P eIF2B (tetrameric subcomplex rotation of 1.6°) (refs. ^13,14,18,32^). Relative to ISRIB-bound A-state eIF2B (ref. ^21^), the δL516A ISRIB pocket widened by 5.2 Å, and the eIF2α-P binding site widened by 2.2 Å (Fig. 4d)^14^. Similarly, compared to the eIF2B–eIF2 structure^18^, the substrate-binding pocket widened by 4.5 Å in δL516A and by 2.1 Å in the eIF2B–eIF2α-P structure (Extended Data Fig. 8)^18^. Close examination of the δL516A switch-helix confirmed that δF443 defaulted to an I-state ‘up’ rotameric position (Fig. 4e and Extended Data Fig. 5d). Critically, in the δL516A structure, δR517 also formed the I-state salt-bridged conformation, reinforcing the notion that the salt bridge and rotamer elements of the switch-helix are functionally coupled.

**Extended Data Fig. 9:**
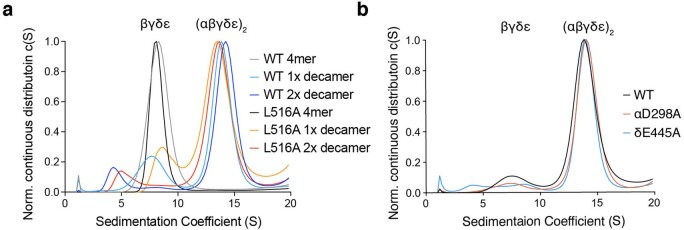
Decamerization propensity of eIF2B Switch-Helix variants. (**a**) Sedimentation velocity analytical ultracentrifugation analysis of WT and δL516A variant eIF2B. ‘1X decamer’ was assembled with a 2:1 stoichiometry of eIF2Bβγδε tetramer:eIF2Bα_2_ dimer. ‘2x decamer’ was assembled with a 1:1 stoichiometry of eIF2B tetramer:eIF2Bα_2_ dimer. Shown is one representative of two replicate experiments. (**b**) Sedimentation velocity analytical ultracentrifugation analysis of eIF2B(αβγδε)_2_ decamers with indicated point mutations. Shown is one representative of three replicate experiments. Source data

To exclude the possibility that the mutations impacted eIF2B decamerization, we analyzed mutant eIF2B complexes by sedimentation velocity analytical ultracentrifugation. We observed no defect in decamerization for δL516A, δF443A, αD298A or δE445A (Extended Data Fig. 9a,b).

### eIF2B switch-helix rearranges after decamer assembly

We next returned to the HDX–MS observation of a selective increase in switch-helix peptide protection after tetramer → decamer assembly. The observation that the degree of protection for the switch-helix was higher than that observed for the surrounding interface that becomes buried during decamer assembly suggested that increased protection arises from local changes in the switch-helix (Fig. 2c).

**Fig. 5:**
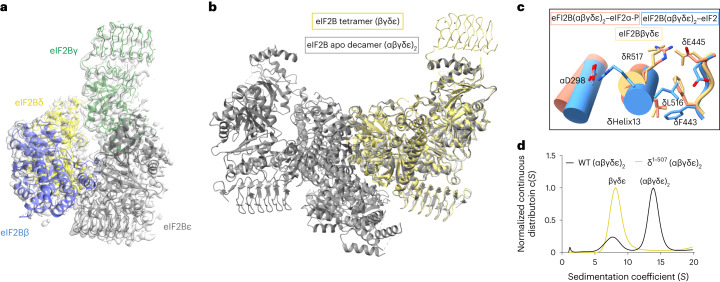
The eIF2B switch-helix is triggered in the tetramer → decamer transition. **a**, Atomic model of the eIF2B tetramer (PDB 6TQO) overlaid with EM density (EMD-41510). **b**, Overlay of the eIF2Bβγδε tetramer structural model onto the apo eIF2B(αβγδε)_2_ decamer structural model (PDB 7L70). **c**, Overlay of the eIF2Bβγδε tetramer switch-helix side chains (yellow) onto the eIF2-bound eIF2B A-state decamer (blue; PDB 6O81) and the eIF2α-P-bound eIF2B I-state (salmon; PDB 6O9Z) atomic models. **d**, Sedimentation velocity analytical ultracentrifugation analysis of WT eIF2B(αβγδε)_2_ and eIF2B(αβγδε)_2_ without the eIF2Bδ C-terminal helix (δ^1–507^). Source data

**Extended Data Fig. 10:**
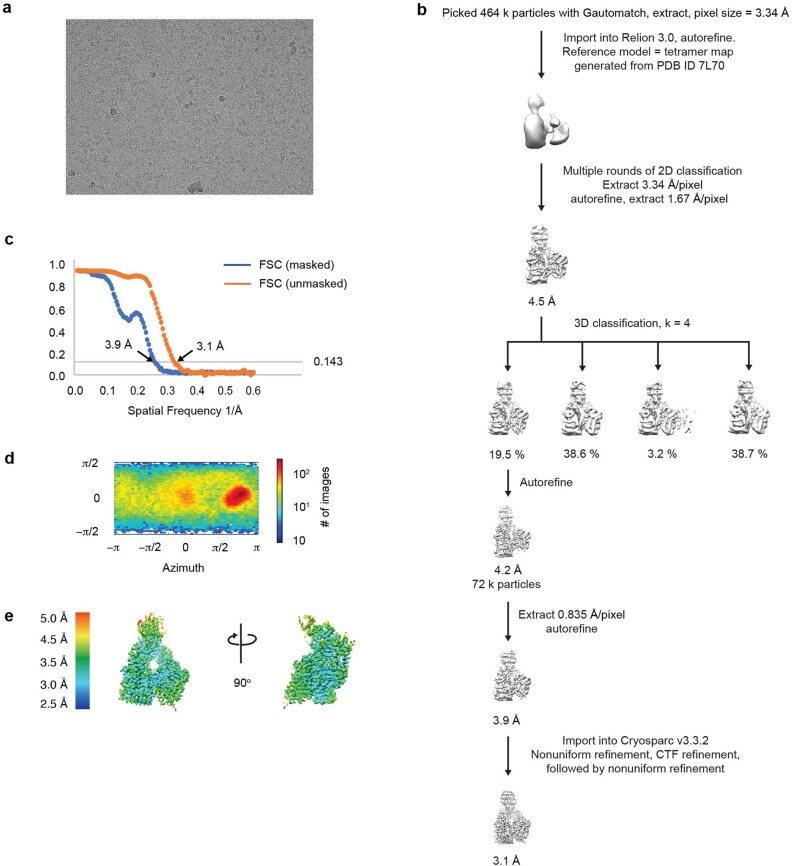
Cryo-EM data analysis of the tetrameric eIF2Bβγδε structure. (**a**) Representative micrograph showing the quality of data used for the final reconstruction of the eIF2Bβγδε structure. A total of 2692 micrographs were acquired and analyzed. (**b**) Data processing scheme of the eIF2Bβγδε structure. (**c**) Fourier shell correlation (FSC) plots of the 3D reconstructions of eIF2Bβγδε unmasked (dark blue), masked (orange). (**d**) Orientation angle distribution of the eIF2Bβγδε reconstruction. (**e**) Local resolution map of the eIF2Bβγδε structure.

To explore conformational change and side chain interactions associated with the assembly reaction in the switch-helix, we determined a cryo-EM structure of the eIF2Bβγδε tetramer to a resolution of 3.1 Å (Fig. 5a, Supplementary Table 3 and Extended Data Fig. 10). Overall, the architecture of the tetramer closely resembles that of the eIF2Bβγδε tetramer in the context of the eIF2B(αβγδε)_2_ decamer structure (Fig. 5b). A close look at the switch-helix elements revealed that the side chains in the eIF2Bβγδε tetramer adopt the I-state conformation, with δR517 forming a salt bridge with δE445 and the δF443 rotamer in the ‘up’ position (Fig. 5c and Extended Data Fig. 5d), consistent with the increased protection seen in HDX–MS.

To explore a possible role of the switch-helix in decamerization, we performed analytical ultracentrifugation experiments on eIF2B mutants in which the switch-helix was truncated (eIF2Bαβγδ^1–507^ε) and found that it was required for decamerization (Fig. 5d). Hence, we conclude that protection of the switch-helix after addition of the eIF2Bα_2_ dimer reflects both decamerization and the adoption of the A-state switch-helix conformation.

### Switch-helix mutations tune integrated stress response signaling in cells

**Fig. 6:**
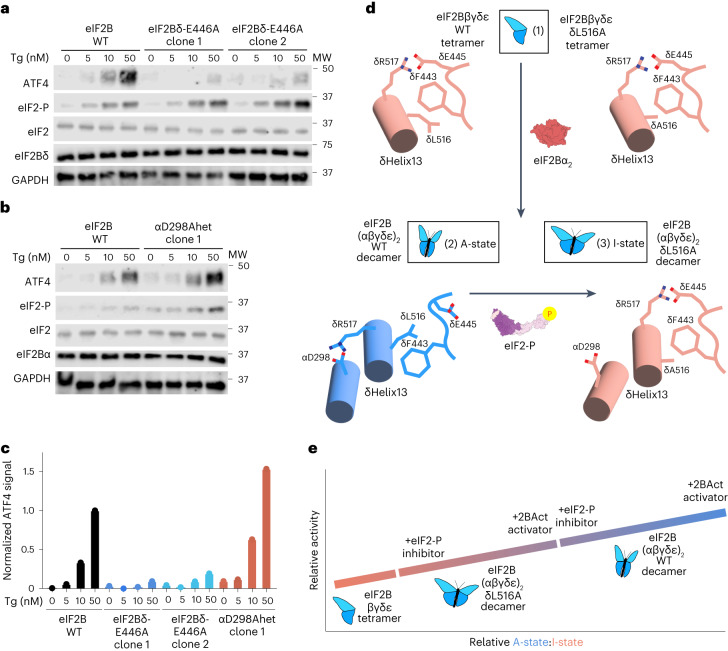
eIF2B switch-helix mutations control ISR signaling in cells. **a**,**b**, AN3-12 mouse ES cells containing *Eif2b4*^E446A^ homozygous (**a**); or **b**) *Eif2b1*^D298A^/*Eif2b1*^WT^ heterozygous (αD298Ahet), endogenously edited mutations were treated with the indicated concentrations of Tg for 1 h and immunoblotted for the indicated proteins. Shown is one representative experiment from a total of three replicates; MW, molecular weight. **c**, Quantitation of ATF4 immunoblot intensity for blots shown in **a** and **b**. The signal was normalized to that of WT 50 nM Tg ATF4 signal. Shown is one representative experiment from a total of three replicates. **d**, The eIF2B switch-helix is in the I-state orientation in the eIF2Bβγδε tetramer (1). Incorporation of eIF2Bα_2_ prompts formation of the δR517–αD298 salt bridge, causing a δL516–δF443 steric clash, triggering conformational change of the switch-helix and global conversion to the eIF2B A-state (2). For the δL516A variant, in the absence of the δL516–δF443 steric clash, the switch-helix does not undergo conformational change, and the global I-state conformation is maintained after decamerization (3). Finally, binding of eIF2-P to apo eIF2B converts the switch-helix from the A-state to the I-state, reverting side chains back to the same I-state arrangement assumed in the tetramer. **e**, Model. eIF2B is in constant equilibrium between I-state and A-state populations. The relative occupancy of A-state and I-state populations can be tuned by the addition of activating effectors such as 2BAct or inhibitory effectors such as eIF2-P inhibitor. Switch-helix mutations can also tune the relative occupancy of A-state and I-state populations. The δL516A mutation shifts the equilibrium toward the I-state, but relative occupancy can still be tuned by effectors. Source data

To determine how switch-helix orientation impacts ISR signaling in cellula, we introduced switch-helix mutations at the endogenous locus of pseudohaploid AN3-12 mouse embryonic stem cells, which were chosen to facilitate ease of precision genome editing. Using CRISPR–Cas9, we engineered three homozygous *Eif2b4*^E446A^ (homologous to human *EIF2B4*^E445A^) clones and two heterozygous *Eif2b1*^D298A^/*Eif2b1*^WT^ clones. Importantly, eIF2Bα and eIF2Bδ protein levels remained comparable to those of the unedited parental cells, demonstrating that the mutation did not destabilize the mutant subunits or disrupt stoichiometry between eIF2B complex members (Fig. 6a,b). Consistent with predictions for A-state stabilization, eIF2Bδ-E446A homozygous clones showed reduced ATF4 induction after treatment with thapsigargin (Tg), a small-molecule inhibitor of the endoplasmic reticulum Ca^2+^ pump and potent inducer of the ISR (Fig. 6a,c). Consistent with expectations of I-state stabilization, αD298A heterozygous clones showed increased induction of ATF4 after Tg treatment (Fig. 6b,c). We did not recover any homozygous αD298A clones, likely because the extreme I-state caused by this mutation induced strong ISR signaling that promoted cell death. Nevertheless, the observation of increased ATF4 induction after heterozygous expression of the αD298A mutation is consistent with the interpretation that cells bearing a mixture of WT eIF2B and αD298A are primed to launch a stronger ISR response than unedited cells (Fig. 6b,c).

## Discussion

Through comprehensive characterization of eIF2B remodeling events via HDX–MS, cryo-EM, biochemistry and cellular signaling experiments, we discovered that eIF2B allosteric coordination is mediated by a switch that is triggered both in A-state → I-state and tetramer → decamer transitions. This conformational switch is orchestrated by a highly conserved helix that is located at the core of the eIF2B decamer and facilitates the hinging motion that converts eIF2B from the A-state to the I-state (Figs. 1a and 3a). Notably, this helix, now referred to as the switch-helix, can be triggered by binding of eIF2B effectors as far as 40 Å away (ISRIB pocket), underlining that it is part of an intersubunit allosteric communication network. Putative components of this network are suggested by shared regions of protection observed in both NSs-bound and 2BAct-bound A-state-biased HDX–MS datasets (Extended Data Fig. 4c–d, yellow).

Switch-helix conformation correlates with eIF2B activity; side chain arrangements are identical in low-activity tetramers and I-state decamers and switch to the opposite state in high-activity A-state decamers. Specifically, in the eIF2B tetramer, the δF443 rotamer is in the ‘up’ position, and δR517 is salt bridged to δE445 (Fig. 6d, step 1). After decamerization to the A-state, αD298 becomes available, and the helix rotates such that δR517 forms a salt bridge to αD298, while δL516 is forced into a position that would clash with the ‘up’ δF443 rotameric state (Fig. 6d, step 2), in turn forcing the δF443 rotamer to adopt the ‘down’ A-state conformation. Binding of eIF2-P inhibitor to induce the I-state decamer reverses the switch to the same position it assumes in the eIF2B tetramer (Fig. 6d, step 3). As such, assembly state and A-state → I-state conformational transitions use shared allosteric machinery.

We propose that the switch-helix acts as a fulcrum within eIF2B to tune ISR signaling. Local changes in switch-helix orientation are propagated to effector binding regions via tuning the relative angle between eIF2Bα and eIF2Bδ subunits (Fig. 4c,d and Extended Data Fig. 8). Switch-helix mutations alter the eIF2B conformational landscape while still enabling regulation by effectors such as eIF2-P (Fig. 3f). Specifically, binding energy lent by switch-helix side chains or eIF2B effectors alters the relative population of the two switch-helix states, enabling fine-tuning of ISR signaling (Fig. 6e).

It has been proposed that apo eIF2B can sample both the I-state and A-state, which is consistent with our observation that switch-helix deuteration rates are similar in apo eIF2B and I-state eIF2B^32^. Assuming that dynamic sampling of the I- and A-states occurs faster than the HDX–MS experimental timescale, the HDX–MS profile would be dominated by the faster exchanging species (the I-state), even if it was not the majority of the population (Fig. 2g). This framework sheds light on previous cryo-EM reports that the stoichiometry of eIF2-P binding affects the severity of eIF2B rotation around the central hinge associated with A-state → I-state conversion^13^. Given the twofold symmetric structure of eIF2B, we propose that binding of a single eIF2-P biases eIF2B toward the I-state to a lesser degree than binding of two eIF2-P molecules. This view is consistent with published cryo-EM models derived from average populations of particles; an increased degree of rotation around the central axis was reported with increased eIF2-P:eIF2B stoichiometric ratio^13^. Notably, the δL516A structure forms a ‘super I-state’, with a wider overall rotation across the central hinge than is observed in eIF2-P-bound structures. One plausible explanation for this increased degree of rotation is that eIF2-P occupancy was not saturated in previously reported cryo-EM structures. Also notable is the fact that on the apo side of the singly bound eIF2B–eIF2-P complex, as determined by cryo-EM, the switch-helix assumes the I-state, consistent with a cross-complex allosteric network^13^.

As a solvent-exposed central fulcrum mediating conformational transitions that tune eIF2B activity, the switch-helix is a promising target for development of novel small-molecule therapeutics. Stabilization of the switch-helix into the A-state would be predicted to have ISRIB-like effects on ISR signaling; indeed, we show that the δE446A mutation programs an ISRIB-like state in cells (Fig. 6a).

Taken together, our work integrates multiple structural and biochemical techniques to identify and interrogate the switch-helix as a central allosteric mediator of the ISR. The switch-helix controls both eIF2B conformational and assembly state transitions by functioning as a molecular fulcrum to affect distant signaling features. HDX–MS enabled its discovery, and cryo-EM structures revealed specific side chain interactions that act as highly conserved allosteric hot spots. Structure-guided switch-helix mutations demonstrated that switch-helix position regulates eIF2B activity and ISR signaling in cells. The atomic-level understanding of eIF2B’s allosteric mechanism cements a new foundation for rational control of ISR signaling and adds dimension to the molecular understanding of allosteric mechanisms in large signaling complexes.

## Methods

### Purification and assembly of human eIF2B subcomplexes

Human WT eIFBα_2_ (pJT075), eIFBα^D298A^_2_ (pRL055), WT eIF2Bβγδε (pJT073 and pJT074 coexpression), eIF2Bβγδε δL516A (pRL036 and pJT074 coexpression), eIF2Bβγδ^F443A^ε (pRL035 and pJT074 coexpression) and eIF2Bβγδ^E445A^ε (pRL049 and pJT074 coexpression) were purified as previously described^21^.

### Purification of viral NSs protein

Viral NSs::6×His was purified as previously described^32^. Briefly, we used the pMS113 construct to express and purify NSs::6×His. Expi293T cells (Thermo Fisher) were transfected with the NSs construct, as per the manufacturer’s instructions, for the MaxTiter protocol and were collected 5 d after transfection. Cells were pelleted (1,000*g*, 4 min) and resuspended in lysis buffer (130 mM KCl, 2.5 mM MgCl_2_, 25 mM HEPES-KOH (pH 7.4), 2 mM EGTA, 1% Triton X-100, 1 mM TCEP and 1× cOmplete protease inhibitor cocktail (Roche)). Cells were then incubated for 30 min at 4 °C and spun at 30,000*g* for 1 h to pellet cell debris. The lysate was applied to a 5-ml HisTrap HP column (GE Healthcare) equilibrated in Buffer A (20 mM HEPES-KOH (pH 7.5), 200 mM KCl, 5 mM MgCl_2_ and 15 mM imidazole) and eluted using a gradient of Buffer B (20 mM HEPES-KOH (pH 7.5), 200 mM KCl, 5 mM MgCl_2_ and 300 mM imidazole). NSs::6×His was concentrated using a 10-kDa molecular weight cutoff spin concentrator (Amicon) and further purified by size-exclusion chromatography over a Superdex 200 Increase 10/300 GL column (GE Healthcare) in elution buffer (20 mM HEPES (pH 7.5), 200 mM KCl, 5 mM MgCl_2_, 1 mM TCEP and 5% glycerol). The resulting fractions were pooled and flash-frozen in liquid nitrogen.

### Purification of heterotrimeric human eIF2

All experiments performed before 1 March 2022 were performed using human eIF2 purified as previously described^33^. Some experiments were performed using purified eIF2 that was a generous gift from Calico Life Sciences. All experiments performed after 1 March 2022 used eIF2 purified according to a second previously described protocol^19^.

### Phosphorylation of eIF2 trimer (eIF2-P) and eIF2α (eIF2α-P)

To generate phosphorylated eIF2 trimers or eIF2α, 25 µM eIF2 trimer or eIF2α was incubated with 500 nM recombinant PERK kinase domain (purified in-house as previously described^18^) and 1 mM ATP at 37 °C for 1 h. Phosphorylation of the final product was verified by 12.5% SuperSep PhosTag gels (Wako Chemical Corporation).

### Assembly of eIF2B decamer complexes

All eIF2B(αβγδε)_2_ used throughout was assembled by mixing purified eIF2Bβγδε and eIF2Bα_2_ at a molar ratio of 2:1.2 eIF2Bβγδε:eIF2Bα_2_ unless otherwise indicated. Complexes were assembled at 10 µM and incubated at 4 °C for 30 min before dilution to experimental conditions.

### Assembly of eIF2B complexes for hydrogen–deuterium exchange–mass spectrometry characterization

For HDX–MS experiments, after preparation of 10 µM eIF2B(αβγδε)_2_, 10× mixtures containing 5 µM eIF2B(αβγδε)_2_ and 12 µM NSs or eIF2-P (representing a 1.2-fold molar excess of NSs and eIF2-P relative to available eIF2B binding sites) were prepared in HDX buffer and allowed to assemble overnight at 4 °C. The tetramer sample was prepared by diluting eIF2Bβγδε to 10 µM. The 10× 2BAct samples were prepared by combining 5 µM eIF2B with 10 µM 2BAct dissolved in DMSO. Matched 10× control samples were prepared by combining 5 µM eIF2B with 2% DMSO such that the final experimental DMSO concentration was 0.2%.

### Hydrogen–deuterium exchange

For all HDX experiments, deuterated buffer was prepared by lyophilizing eIF2B assay buffer (20 mM HEPES, 200 mM KCl, 5 mM MgCl_2_ and 1 mM TCEP, pH 7.9) and resuspending it in deuterium oxide (Sigma-Aldrich, 151882). To initiate the continuous-labeling experiment, samples were diluted tenfold to 1× (final eIF2B tetramer concentration of 1 µM or a final eIF2B decamer concentration of 0.5 µM) into temperature-equilibrated deuterated eIF2B assay buffer. Samples were quenched at the time points outlined below by mixing 30 μl of the partially exchanged protein with 30 μl of 2× quench buffer (6 M urea and 500 mM TCEP, pH 2.4) on ice. Samples were incubated on ice for 1 min to allow for partial unfolding to assist with proteolytic degradation and were then flash-frozen in liquid nitrogen and stored at −80 °C. The HDX time points for these experiments were 10 s, 100 s, 15 min and 3 h.

### Protease digestion and liquid chromatography–mass spectrometry

All samples were thawed immediately before injection into a cooled valve system (Trajan LEAP) coupled to an LC (Thermo UltiMate 3000). Sample time points were injected in random order. The temperature of the valve chamber, trap column and analytical column was maintained at 2 °C. The temperature of the protease column was maintained at 10 °C. The quenched sample was subjected to in-line digestion by two immobilized acid proteases in order, aspergillopepsin (Sigma-Aldrich, P2143) and porcine pepsin (Sigma-Aldrich, P6887), at a flow rate of 200 μl min^–1^ of Buffer A (0.1% formic acid). Protease columns were prepared in-house by coupling protease to beads (Thermo Scientific POROS 20 AL aldehyde activated resin, 1602906) and packed by hand into a column (2 mm (inner diameter) × 2 cm, IDEX C-130B). Following digestion, peptides were desalted for 4 min on a hand-packed trap column (Thermo Scientific POROS R2 reversed-phase resin, 1112906, 1 mm (inner diameter) × 2 cm, IDEX C-128). Peptides were then separated with a C8 analytical column (Thermo Scientific BioBasic-8, 5-μm particle size, 0.5 mm (inner diameter) × 50 mm, 72205-050565) and a gradient of 5–40% Buffer B (100% acetonitrile and 0.1% formic acid) at a flow rate of 40 μl min^–1^ over 14 min and then a gradient of 40–90% Buffer B over 30 s. The analytical and trap columns were then subjected to a sawtooth wash and equilibrated at 5% Buffer B before the next injection. Protease columns were washed with two injections of 100 μl of 1.6 M guanidinium chloride and 0.1% formic acid before the next injection. Peptides were eluted directly into a Q Exactive Orbitrap mass spectrometer operating in positive mode (resolution of 70,000, automatic gain control target of 3 × 10^6^, maximum injection time of 50 ms and scan range of 300–1,500 *m*/*z*). For each eIF2B condition, a tandem MS experiment was performed (full MS settings were the same as described above, and data-dependent MS^2^ settings included a resolution of 17,500, automatic gain control target of 2 × 10^5^, maximum injection time of 100 ms, loop count of 10, isolation window of 2.0 *m*/*z*, normalized collision energy of 28, charge state of 1 and ≥7 excluded and dynamic exclusion of 15 s) on undeuterated samples. LC and MS methods were run using Xcalibur 4.1 (Thermo Scientific).

### Peptide identification

Byonic (Protein Metrics) was used to identify peptides in the tandem MS data. Sample digestion parameters were set to nonspecific. Precursor mass tolerance and fragment mass tolerance were set to 6 and 10 ppm, respectively. Peptide lists (sequence, charge state and retention time) were exported from Byonic and imported into HDExaminer 3 (Sierra Analytics). When multiple peptide lists were obtained, all were imported and combined in HDExaminer 3.

### HDExaminer 3 analysis

Peptide isotope distributions at each exchange time point were fit in HDExaminer 3. Deuteration levels were determined by subtracting mass centroids of deuterated peptides from undeuterated peptides. All peptides we monitored showed EX2 behavior.

### Hydrogen–deuterium exchange data presentation

We define a notable increase or decrease of deuteration as at least three peptides with a change in number of deuterons of >0.5. This was determined after measuring the average noise in our dataset; for biological replicates run on the same day on the instrument, the average standard deviation for the number of deuterons taken up was 0.075. We set a conservative threshold of at least three peptides over 0.5 change in number of deuterons, which represents 6.7 standard deviations.

### BODIPY-GDP exchange assay

In vitro detection of GDP binding to eIF2 was adapted from a published protocol for a fluorescence intensity-based assay describing dissociation of eIF2 and nucleotide^34^. We first performed a loading assay for fluorescent BODIPY-FL-GDP as previously described^21^. Purified eIF2 (137.5 nM) was incubated with 100 nM BODIPY-FL-GDP (Thermo Fisher Scientific) in assay buffer (100 mM HEPES-KOH (pH 7.5), 100 mM KCl, 5 mM MgCl_2_, 1 mM TCEP and 1 mg ml^–1^ bovine serum albumin) to a volume of 500 µl in a black-walled 1.5-ml tube. This mix was then added to 384-square-well, black-walled, clear-bottom polystyrene assay plates (Corning, 3766) with 18 µl per well. A GEF mix composed of a 10× solution of eIF2B(αβγδε)_2_ was prepared. To compare nucleotide exchange rates, 2 µl of the 10× GEF mixes was spiked into the 384-well plate wells with a multichannel pipette, such that the resulting final concentration of eIF2B(αβγδε)_2_ was 5 nM, and the final concentrations of other proteins and drugs are as indicated in the figures. Subsequently, in the same wells, we performed a ‘GDP unloading assay’ as indicated in the figures. After completion of the loading reaction, wells were spiked with 1 mM GDP to start the unloading reaction at *t* = 0. In the case of inhibition assays with eIF2-P, the eIF2/BODIPY-GDP mix was also incubated with 25 nM eIF2B(αβγδε)_2_, and a 10× mix of eIF2-P was spiked into the wells so that the final concentration was 50 nM. For all GEF assays involving eIF2-P, an ‘unloading’ assay was used because the eIF2B(αβγδε)_2_ had been preincubated with eIF2. Fluorescence intensity was recorded every 10 s for 60 min at 25 °C using a Clariostar Plus (BMG LabTech) plate reader (excitation wavelength of 497 nm and bandwidth of 14 nm; emission wavelength of 525 nm and bandwidth of 30 nm). Data collected were fit to a first-order exponential.

### FAM-ISRIB binding assay

All fluorescence polarization measurements were performed in 20-μl reactions with 100 nM eIF2B(αβγδε)_2_ + 2.5 nM FAM-ISRIB (Praxis Bioresearch) in FP buffer (20 mM HEPES-KOH (pH 7.5), 100 mM KCl, 5 mM MgCl_2_ and 1 mM TCEP) and measured in 384-well, non-stick black plates (Corning, 3820) using a ClarioStar Plus (BMG LabTech) plate reader at room temperature. Before the reaction setup, eIF2B(αβγδε)_2_ was assembled in FP buffer using eIF2Bβγδε and eIF2Bα_2_ in a 2:1.5 molar ratio for at least 1 h at 4 °C. FAM-ISRIB was always first diluted to 2.5 μM in 100% NMP before dilution to 50 nM in 2% NMP and then added to the reaction. For titrations with eIF2α and eIF2α-P, dilutions were made in FP buffer. For titrations with ISRIB, ISRIB was first diluted with 100% NMP to 2.5 µM and then to the final concentrations in 4% NMP. The reactions with eIF2B, FAM-ISRIB and the dilutions were incubated at 25 °C for 30 min before measurement of parallel and perpendicular intensities (excitation of 482 nm and emission of 530 nm).

### FAM-ISRIB kinetic binding assay

The kinetic characterization of FAM-ISRIB binding during eIF2α phosphorylation was assayed in 18-μl reactions of 100 nM eIF2B(αβγδε)_2_, 2.5 nM FAM-ISRIB, 100 μM ATP and 5.6 μM eIF2α/eIF2α-P in FP buffer. These solutions were preincubated at 22 °C for 30 min before polarization was measured every 15 s (30 flashes per s). After four cycles, 2 μl of homemade PERK kinase domain at 0.1 mg ml^–1^ was added for a final concentration of 10 μg ml^–1^ in the reaction, and measurement was resumed for 1 h.

### Structural measurements

Measurement of rotation of the switch-helix between the A-state and I-state was performed by comparing the position of the first Cα away from the amide backbone of PDB 6O81 (eIF2-bound eIF2B) to that of PDB 6O9Z (eIF2α-P-bound eIF2B). For global rotational changes in Fig. 4, the hinge movement between the two eIF2B halves was measured between the lines connecting eIF2Bε H352 and P439.

### Analytical ultracentrifugation

Analytical ultracentrifugation sedimentation velocity experiments were performed as previously described using the ProteomeLab XL-I system (Beckman Coulter)^21^. Briefly, samples were loaded into cells in a buffer consisting of 20 mM HEPES-KOH (pH 7.5), 150 mM KCl, 1 mM TCEP and 5 mM MgCl_2_. A buffer-only reference control was also loaded. Samples were then centrifuged in an AN-50 Ti rotor at 40,000 r.p.m. at 20 °C, and 280-nm absorbance was monitored. Subsequent data analysis was conducted with Sedfit using a non-model-based continuous c(*S*) distribution.

### Sample preparation for cryo-electron microscopy

Decameric eIF2Bδ δL516A was prepared by incubating 20 μM eIF2Bδ^L516A^ βγδε with 11 µM eIF2Bα_2_ in a final solution containing 20 mM HEPES-KOH, 200 mM KCl, 5 mM MgCl_2_ and 1 mM TCEP. eIF2Bδ δL516A decamers and eIF2B tetramers were diluted to 750 nM in 20 mM HEPES-KOH, 200 mM KCl, 5 mM MgCl_2_ and 1 mM TCEP before grid application. For grid freezing, a 3-μl aliquot of the sample was applied to a Quantifoil R1.2/1/3 400-mesh gold grid, followed by a 30-s waiting period. A 0.5-μl aliquot of 0.1–0.2% Nonidet P-40 substitute was added immediately before blotting. The entire blotting procedure was performed using Vitrobot (FEI) at 10 °C and 100% humidity.

### Electron microscopy data collection

Cryo-EM data were collected on a Titan Krios transmission electron microscope operating at 300 keV. Micrographs were acquired using a Gatan K3 direct electron detector. The total dose was 67 e^–^ Å^–2^, and 117 frames were recorded during a 5.9-s exposure. Data were collected at 105,000× nominal magnification (0.835 Å per pixel at the specimen level), with a nominal defocus range of −0.6 to −2.0 μm.

### Image processing

The micrograph frames were aligned using MotionCor2 (ref. ^35^). The contrast transfer function (CTF) parameters were estimated with GCTF^36^. For the decameric eIF2Bδ δL516A, particles were picked in Cryosparc v3.3.2 using apo eIF2B (Electron Microscopy Data Bank (EMDB) EMD-23209) as a template^14,37^. Particles were extracted using an 128-pixel box size and were classified in 2D. Classes that showed clear protein features were selected and extracted for heterogeneous refinement using models of an apo decamer, a tetramer and an impurity class, followed by homogenous refinement. Particles belonging to the decamer class were then reclassified using heterogeneous refinement to sort the best resolution class. Particles from the resulting best class were then reextracted with a pixel size of 0.835 Å and subjected to nonuniform refinement, yielding a reconstruction of 3.0 Å. These particles were subjected to CTF refinement to correct for the per-particle CTF and beam tilt. A final round of nonuniform refinement yielded the final structure of 2.9 Å.

For the tetramer structure, particles were picked by Gautomatch and extracted at a pixel size of 3.34 Å per pixel. Particles were imported into Relion 3.0 for autorefinement to generate a consensus structure. These particles were then subjected to multiple rounds of 2D classification, where particles that represent proteins were selected and reextracted. The resulting set of particles were subjected to autorefinement, followed by reextraction at 1.67 Å per pixel and another round of autorefinement, yielding a reconstruction at 4.5 Å. These particles were then subjected to 3D classification (*k* = 4), and the best class was selected for further refinement, which generated a 4.2-Å reconstruction. Particles belonging to this set (~72,000) were extracted at 0.835 Å per pixel and subjected to autorefinement, yielding a 3.9-Å structure. Particles belonging to this class were imported into Cryosparc v3.3.2, where they were subjected to nonuniform refinement and CTF refinement to yield the final structure at a resolution of 3.1 Å.

### Atomic model building, refinement and visualization

For both the decamer and the tetramer structures, the previously published apo eIF2B model (PDB 7L70) was used as a starting model^14^. Each subunit was docked into the EM density individually and subjected to rigid body refinement in Phenix^38^. The models were then manually adjusted in Coot and refined in phenix.real_space_refine using global minimization, secondary structure restraints, Ramachandran restraints and local grid search^39^. Iterative cycles of manual rebuilding in Coot and phenix.real_space_refine were then performed. The final model statistics were tabulated using Molprobity^40^. Molecular graphics and analyses were performed with the University of California San Francisco (UCSF) Chimera package. UCSF Chimera is developed by the Resource for Biocomputing, Visualization and Informatics and is supported by NIGMS P41-GM103311. The atomic model is deposited at PDB under the accession codes 8TQZ (eIF2Bδ δL516A) and 8TQO (tetramer). The EM map is deposited at EMDB under the accession codes EMD-41566 (eIF2Bδ δL516A) and EMD-41510 (tetramer).

### Generation of endogenously edited cells

Editing of the *Eif2b1* and *Eif2b4* genes to introduce D298A and E446A mutations, respectively, into mouse AN3-12 pseudohaploid embryonic stem cells obtained from the Austrian Haplobank (https://haplobank.org) was performed using nucleofection of CRISPR–Cas9 ribonucleoproteins, as previously described (https://www.protocols.io/view/cas9-sgrna-ribonucleoprotein-nucleofection-using-l-261ge1xyv479/v10) using single guide RNAs and single-stranded homology-directed repair templates listed in Supplementary Table 4. Nucleofection was performed using a 4D-Nucleofector with X-unit attachment (Lonza) and with pulse program CG-104. Two days after nucleofection, genomic DNA was extracted using a PureLink Genomic DNA mini kit from a portion of cells, relevant genes were PCR amplified, and editing efficiency was determined using the Synthego ICE tool (https://ice.synthego.com/#/). When editing efficiency was confirmed to be at least 10%, cells were diluted to an expected density of 0.0625 cells per well, plated onto 96-well plates and allowed to grow up from single colonies, with media changes every 3–5 d depending on the media acidification rate. Genomic DNA was extracted from colonies derived from single clones, and successful editing was determined by PCR amplification of the gene of interest and analysis using the Synthego ICE tool. All cell lines were negative for *Mycoplasma* contamination.

### Western blotting

Cells were seeded at 1 × 10^6^ cells per well of a six-well plate and grown at 37 °C and 5% CO_2_ for 24 h. Cells were treated using the indicated Tg concentrations (Invitrogen) for 1 h, ensuring that the final DMSO concentration was 0.1% across all conditions. Plates were put on ice, and cells were washed once with ice-cold PBS and lysed in 200 μl of ice-cold lysis buffer (50 mM Tris-HCl (pH 7.4), 150 mM NaCl, 1 mM EDTA, 1% (vol/vol) Triton X-100, 10% (vol/vol) glycerol, 1× cOmplete protease inhibitor cocktail (Roche) and 1× PhosSTOP (Roche)). Cells were scraped, collected in an Eppendorf tube and rotated for 30 min at 4 °C. Debris was pelleted at 12,000*g* for 10 min at 4 °C, and the supernatant was removed to a new tube on ice. Protein concentration was normalized to 15 µg of total protein per lane using Bio-Rad Protein Assay Dye. A 5× Laemmli loading buffer (250 mM Tris-HCl (pH 6.8), 30% glycerol, 0.25% bromophenol blue, 10% SDS and 5% β-mercaptoethanol) was added to each sample to 1×, and samples were denatured at 95 °C for 5 min and spun down. Wells of AnyKd Mini-Protean TGX precast protein gels (Bio-Rad) were loaded with equal amounts of total protein in between Precision Plus Dual Color protein ladder (Bio-Rad). After electrophoresis, proteins were transferred onto a nitrocellulose membrane and blocked for 2 h at room temperature in PBS with 0.1% Tween + 3% milk (blocking buffer) while rocking. Primary antibody staining was performed with gentle agitation at 4 °C overnight using the conditions outlined in Supplementary Table 3. After washing four times in appropriate blocking buffer, secondary antibody staining was performed for 1 h at room temperature using anti-rabbit horseradish peroxidase or anti-mouse horseradish peroxidase (Promega, 1:10,000) in blocking buffer. Membranes were washed three times in blocking buffer and then one time in PBS with 0.1% Tween without milk. Membranes were incubated with SuperSignal West Dura or Femto (Thermo Fisher Scientific) for 5 min. Membranes were imaged on a LI-COR Odyssey gel imager for 0.5–10 min depending on band intensity.

### Reporting summary

Further information on research design is available in the Nature Portfolio Reporting Summary linked to this article.

## Online content

Any methods, additional references, Nature Portfolio reporting summaries, source data, extended data, supplementary information, acknowledgements, peer review information; details of author contributions and competing interests; and statements of data and code availability are available at 10.1038/s41589-023-01453-9.

### Supplementary information

Supplementary Information Supplementary Tables 1–4.

Reporting Summary

Supplementary Video 1 Visualization of eIF2B conformational and switch-helix transformation. Morph between A-state eIF2B (PDB 6O81) and I-state eIF2B (PDB 6O9Z) with only eIF2B chains shown. Switch-helix side chain residues are visualized in red.

Supplementary Data Raw HDExaminer 3 files showing analyzed replicate data for all HDX–MS experiments.

### Source data

Source Data Fig. 1 and Extended Data Fig. 2 HDX–MS peptide uptake data.

Source Data Fig. 2 and Extended Data Fig. 4 HDX–MS peptide uptake data.

Source Data Fig. 3 Raw data from biochemical assays.

Source Data Fig. 4 Raw data from biochemical assays.

Source Data Fig. 5 Raw data from biochemical assays.

Source Data Fig. 6 Uncropped blots corresponding to Fig. 6.

Source Data Extended Data Fig. 9 Raw analytical ultracentrifugation data.

## Data Availability

The data that support this study are available from the corresponding authors upon reasonable request. The cryo-EM structural maps and models generated in this study have been deposited to the PDB (accession codes for the δL516A decamer: PDB ID 8TQZ, EMDB ID EMD-41566; accession codes for the eIF2B tetramer: PDB ID 8TQO, EMDB ID EMD-41510). Source data are provided with this paper.

## Code availability

HDX deuteration summary plots were prepared using Jupyter notebooks in Python. The code is available upon request.
