# Supplementary material for: A helical fulcrum in eIF2B coordinates allosteric regulation of stress signaling
Source: Nat Chem Biol. 2023 Nov 9;20(4):422–31. doi: 10.1038/s41589-023-01453-9 (PMC10972756; doi:10.1038/s41589-023-01453-9)
Supplement: Supplementary file 1 — Supplementary Tables 1–4. [file 41589_2023_1453_MOESM1_ESM.pdf]

nature chemical biology

Article

https://doi.org/10.1038/s41589-023-01453-9

A helical fulcrum in eIF2B coordinates allosteric regulation of stress signaling

In the format provided by the authors and unedited

Supplemental Table 1: HDX-MS data

Dataset	Apo eIF2B paired with NSs and eIF2P	eIF2B + NSs	eIF2B + eIF2-P	Tetra-meric eIF2B	Apo eIF2B decamer with 2x eIF2Bα2, (paired with tetramer)	Apo eIF2B + DMSO	Apo eIF2B + 2BAct (in DMSO)
**HDX reaction details**	Exchange occurred at 25C in 90% D_2O assay buffer (see methods), and quenched via 2-fold dilution into ice-cold pH 2.4 quench buffer (see methods). Samples were incubated on ice 60s then flash-frozen.						
**HDX time course**	0s (undeuterated), 10s, 100s, 15 min, 3 hr						
**HDX controls**	Controls included undeuterated samples as well as performance of enzymatic activity assays to validate protein state prior to data collection.						
**Back-exchange**	Samples were not back-exchange corrected.						
**Number of peptides**	971	971	971	904	904	1056	1056
**Sequence coverage**	96.5%	96.5%	96.5%	93.2%	93.2%	94.2%	94.2%
**Average peptide length/redundancy**	Avg peptide length: 15.1<br><br>Redundancy: 6.2	Avg peptide length: 15.1<br><br>Redundancy: 6.2	Avg peptide length: 15.1<br><br>Redundancy: 6.2	Avg peptide length: 14.0<br><br>Redundancy: 6.1	Avg peptide length: 14.0<br><br>Redundancy: 6.1	Avg peptide length: 14.5<br><br>Redundancy: 6.5	Avg peptide length: 14.5<br><br>Redundancy: 6.5
**Replicates**	3	3	3	3	3	3	3
**Repeatability**	SD of #Deuterons from 3 biological replicates of 2BAct eIF2B decamer samples (all timepoints)= 0.075 deuterons						

Supplemental Table 2: Cryo-EM data collection, analysis, and model building.

Structure	$eIF2B^{\delta L516A}$ (PDB ID: 8TQZ)	eIF2Bβγδε (PDB ID: 8TQO)
**Data collection**		
Microscope	Titan Krios	
Voltage (keV)	300	
Nominal magnification	105000x	
Exposure navigation	Image shift	
Electron dose ($e^{-}Å^{-2}$)	67	
Dose rate (e^{-}/pixel/sec)	8	
Detector	K3 summit	
Pixel size (Å)	0.835	
Defocus range (μm)	0.6-2.0	
Micrographs	4042	2692
**Reconstruction**		
Total extracted particles (no.)	2729647	464356
Final particles (no.)	326478	71704
Symmetry imposed	C1	C1
FSC average resolution, masked (Å)	2.9	3.1
FSC average resolution, unmasked (Å)	3.7	3.9
Applied B-factor (Å)	89.5	115.4
Reconstruction package	Cryosparc v3.3.2	Relion 3 and Cryosparc v3.3.2
**Refinement**		
Protein residues	2866	1437
Ligands	0	0
RMSD Bond lengths (Å)	0.002	0.002
RMSD Bond angles (°)	0.497	0.539
Ramachandran outliers (%)	0.00	0.00
Ramachandran allowed (%)	3.60	4.52
Ramachandran favored (%)	96.40	95.48
Poor rotamers (%)	4.38	0.08
CaBLAM outliers (%)	1.89	2.29
Molprobity score	2.05	1.79
Clash score (all atoms)	5.95	8.77
B-factors (protein)	85.05	111.76
B-factors (ligands)	N/A	N/A
EMRinger Score	2.05	1.75
Refinement package	Phenix 1.17.1-3660-000	Phenix 1.17.1-3660-000

Supplemental Table 3: Western blotting primary antibody conditions

Antibody target	Host	Dilution	Manufacturer	Cat. number	Blocking Conditions
eIF2Bα	Rabbit	1:1000	ProteinTech	18010-1-AP	PBS-T + 3% milk
eIF2Bδ	Rabbit	1:1000	ProteinTech	11332-1-AP	PBS-T + 3% milk
ATF4	Rabbit	1:1000	Cell Signaling	11815S	PBS-T + 3% milk
GAPDH	Rabbit	1:2000	Abcam	ab9485	PBS-T + 3% milk
phospho S51-EIF2S1 (α)	Rabbit	1:1000	Cell Signaling	3398	PBS-T + 3% BSA

Supplemental Table 4: sgRNA and HDRT sequences

Edit	sgRNA sequence	HDRT sequence
eIF2B1 D298A	AGCUCAUCGCUUACAGCAGA	TGGACTACACCTCCCCATCCCTCATCACCCTGCTGTTTACGGACCTGGGTGTGTTGACGCCATCTGCTGTATCCGCTGAGCTCATCAAGCTGTACCTATGAGCCGAGTCGCTCCTGCCAACATGCAGGAAGCACGCGCTGTGCAGGATGAG
eIF2B4 E446A	ACCUAGCUCGUUGGAGACAA	CTTTGGTACCTGGAAGCCTCCGTTCTGCTCTGCTTACCTAGCTCGTTGGAGACAAATGCATCAGTCTGCACACGTGCACAGAACTTGTACGTTTCACAGCAGACCAGTACTGGAACGTTATGAGCTCGAGCCACCAGGGCCAACTGTGCTG
