# Supplementary material for: A helical fulcrum in eIF2B coordinates allosteric regulation of stress signaling
Source: Nat Chem Biol. 2023 Nov 9;20(4):422–31. doi: 10.1038/s41589-023-01453-9 (PMC10972756; doi:10.1038/s41589-023-01453-9)
Supplement: Supplementary file 2 — Reporting Summary [file 41589_2023_1453_MOESM2_ESM.pdf]

nature portfolio

Corresponding author(s): Rosalie Lawrence, Lan Wang, Susan Marqusee, Peter Walter

Last updated by author(s): Aug 23, 2023

Reporting Summary

Nature Portfolio wishes to improve the reproducibility of the work that we publish. This form provides structure for consistency and transparency in reporting. For further information on Nature Portfolio policies, see our Editorial Policies and the Editorial Policy Checklist.

Statistics

For all statistical analyses, confirm that the following items are present in the figure legend, table legend, main text, or Methods section.

n/a	Confirmed	
☐	☒	The exact sample size (*n*) for each experimental group/condition, given as a discrete number and unit of measurement
☐	☒	A statement on whether measurements were taken from distinct samples or whether the same sample was measured repeatedly
☒	☐	The statistical test(s) used AND whether they are one- or two-sided<br>*Only common tests should be described solely by name; describe more complex techniques in the Methods section.*
☒	☐	A description of all covariates tested
☒	☐	A description of any assumptions or corrections, such as tests of normality and adjustment for multiple comparisons
☐	☒	A full description of the statistical parameters including central tendency (e.g. means) or other basic estimates (e.g. regression coefficient) AND variation (e.g. standard deviation) or associated estimates of uncertainty (e.g. confidence intervals)
☒	☐	For null hypothesis testing, the test statistic (e.g. *F*, *t*, *r*) with confidence intervals, effect sizes, degrees of freedom and *P* value noted<br>*Give P values as exact values whenever suitable.*
☒	☐	For Bayesian analysis, information on the choice of priors and Markov chain Monte Carlo settings
☒	☐	For hierarchical and complex designs, identification of the appropriate level for tests and full reporting of outcomes
☒	☐	Estimates of effect sizes (e.g. Cohen's *d*, Pearson's *r*), indicating how they were calculated

Our web collection on statistics for biologists contains articles on many of the points above.

Software and code

Policy information about availability of computer code

Data collection	Detailed in Methods Section as Appropriate
Data analysis	Detailed in Methods Section as Appropriate. The following software versions were used:<br>HDExaminer version 3<br>Motioncor MotionCorr2 v1.3.0<br>Relion: relion 3.0.8<br>Gautomatch_v0.56_sm53_cu8.0<br>Phenix 1.17.1-3660<br>Coot 0.8.9.2<br>Molprobity: version embedded in Phenix 1.17.1-3660<br>Cryosparc: ??

For manuscripts utilizing custom algorithms or software that are central to the research but not yet described in published literature, software must be made available to editors and reviewers. We strongly encourage code deposition in a community repository (e.g. GitHub). See the Nature Portfolio guidelines for submitting code & software for further information.

Data

Policy information about availability of data

All manuscripts must include a data availability statement. This statement should provide the following information, where applicable:

- Accession codes, unique identifiers, or web links for publicly available datasets
- A description of any restrictions on data availability
- For clinical datasets or third party data, please ensure that the statement adheres to our policy

The data that support this study are available from corresponding authors upon reasonable request. The cryo-EM structural maps and models generated in this study have been deposited to the protein data bank (accession codes for L516A eIF2B decamer: PDB ID 8TQZ, EMDB ID EMD-41566; accession codes for eIF2B tetramer: PDB ID 8TQO, EMDB ID EMD-41510). HX-MS data are provided as source data files with this paper.

Human research participants

Policy information about studies involving human research participants and Sex and Gender in Research.

Reporting on sex and gender	N/A
Population characteristics	N/A
Recruitment	N/A
Ethics oversight	N/A

Note that full information on the approval of the study protocol must also be provided in the manuscript.

Field-specific reporting

Please select the one below that is the best fit for your research. If you are not sure, read the appropriate sections before making your selection.

☒ Life sciences ☐ Behavioural & social sciences ☐ Ecological, evolutionary & environmental sciences

For a reference copy of the document with all sections, see nature.com/documents/nr-reporting-summary-flat.pdf

Life sciences study design

All studies must disclose on these points even when the disclosure is negative.

Sample size	For experiments presenting averaged data (median), a minimum of n = 3 replicates was performed unless otherwise stated. This number was selected because is it standard in the HDX-MS field. We determined this to be sufficient owing to low observed variability between replicates.
Data exclusions	During manual curation of data in HDExaminer, low quality peptides (peptides with low signal intensity or peptides that have inconsistent monoisotopic masses) are removed. To be included in the manuscript analysis of continuous labeling experiments, peptides must have triplicate data for each time point, and the deuterium uptake at each time point must have a standard deviation below 1 deuteron.
Replication	Continuous labeling hydrogen exchange experiments were performed in technical triplicate with standard deviations reported in peptide uptake plots. No replication issues were observed.
Randomization	For every condition, all time points (i.e. 10s, 100s, 900s, 14400s) were injected in random order to ensure that observations were not the result of instrument variation.
Blinding	No blinding was conducted during this study.

Reporting for specific materials, systems and methods

We require information from authors about some types of materials, experimental systems and methods used in many studies. Here, indicate whether each material, system or method listed is relevant to your study. If you are not sure if a list item applies to your research, read the appropriate section before selecting a response.

Materials & experimental systems

n/a	Involved in the study
☐	☒ Antibodies
☐	☒ Eukaryotic cell lines
☒	☐ Palaeontology and archaeology
☒	☐ Animals and other organisms
☒	☐ Clinical data
☒	☐ Dual use research of concern

Methods

n/a	Involved in the study
☒	☐ ChIP-seq
☒	☐ Flow cytometry
☒	☐ MRI-based neuroimaging

Antibodies

Antibodies used

All antibodies are detailed in Supplemental Table 3 and below:
Supplemental Table 3: Western blotting primary antibody conditions

Antibody target Host Dilution Manufacturer Cat. number Blocking Conditions
eIF2Bα Rabbit 1:1000 ProteinTech 18010-1-AP PBS-T + 3% milk
eIF2Bδ Rabbit 1:1000 ProteinTech 11332-1-AP PBS-T + 3% milk
ATF4 Rabbit 1:1000 Cell Signaling 11815S PBS-T + 3% milk
GAPDH Rabbit 1:2000 Abcam ab9485 PBS-T + 3% milk
phospho S51-EIF2S1 (α) Rabbit 1:1000 Cell Signaling 3398 PBS-T + 3% BSA

Validation

All antibodies were validated by the manufacturers. In addition, we validated antibody specificity in the following ways depending on recognized protein. For all eIF2B and eIF2 antibodies, western blots of purified proteins were run. For and ATF4, ISR activation was used to confirm specificity of these agents. For details see:

Schoof M, Boone M, Wang L, Lawrence R, Frost A, Walter P. eIF2B conformation and assembly state regulate the integrated stress response. Elife 2021;10:e65703. https://doi.org/10.7554/eLife.65703.

Eukaryotic cell lines

Policy information about cell lines and Sex and Gender in Research

Cell line source(s)

EXPI293F (ThermoFisher cat. A14527), AN3-12 mouse ES cells (provided by Austrian Haplobank: https://www.haplobank.at)

Authentication

EXPI293F were purchased commercially and not further validated; AN3-12 mouse ES cells were received from the Austrian Haplobank, which performs routine karyotyping and visual inspection of morphology.

Mycoplasma contamination

All cell lines tested negative for mycoplasma contamination.

Commonly misidentified lines
(See ICLAC register)

No commonly misidentified lines were used in this study.
