# Supplementary figures and images for: A helical fulcrum in eIF2B coordinates allosteric regulation of stress signaling
Source: Nat Chem Biol. 2023 Nov 9;20(4):422–31. doi: 10.1038/s41589-023-01453-9 (PMC10972756; doi:10.1038/s41589-023-01453-9)

**Fig. 6 Source Data: Unprocessed Gels**

**a**

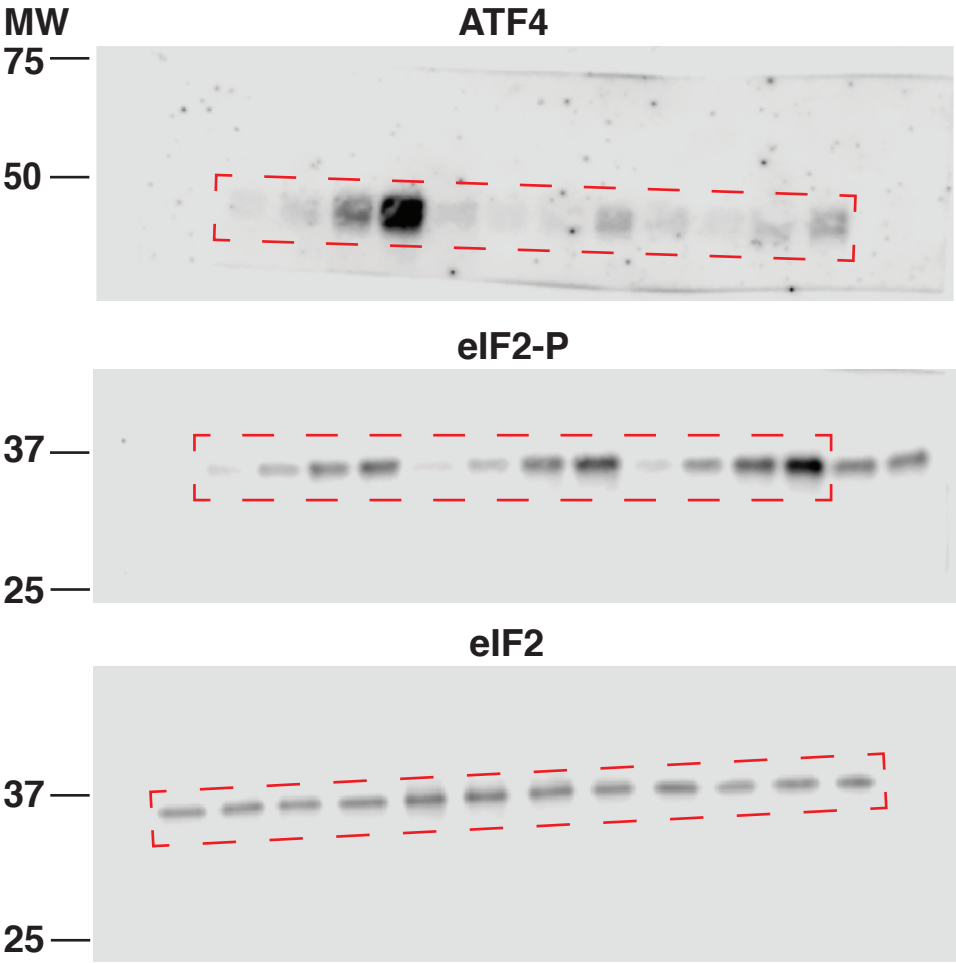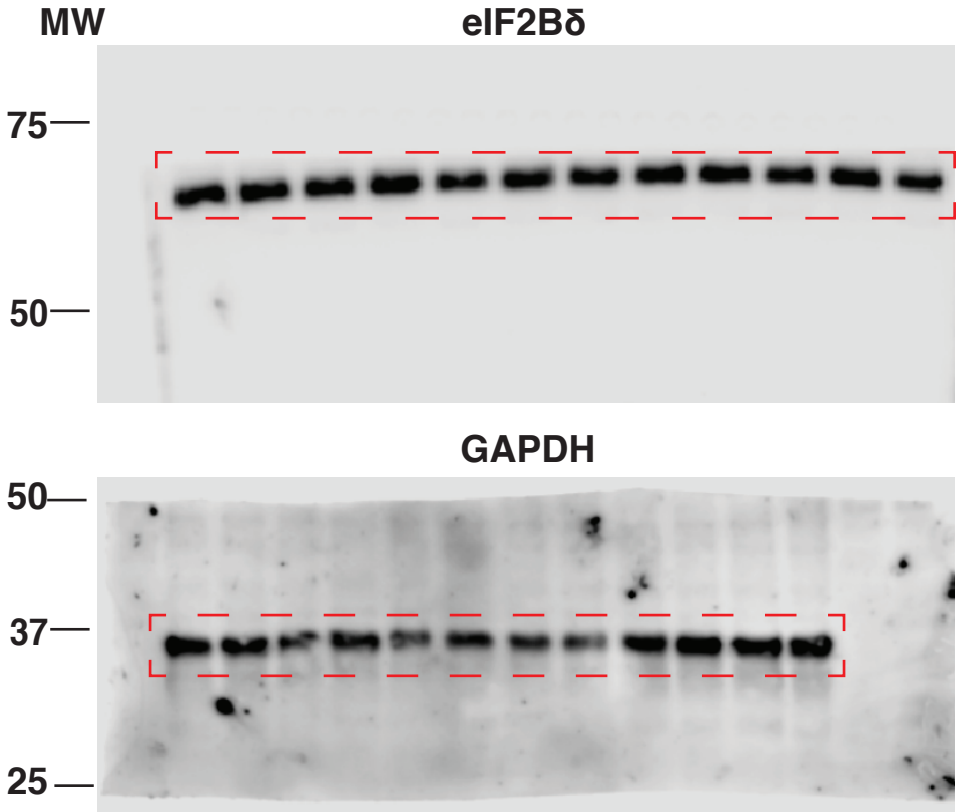

**b**

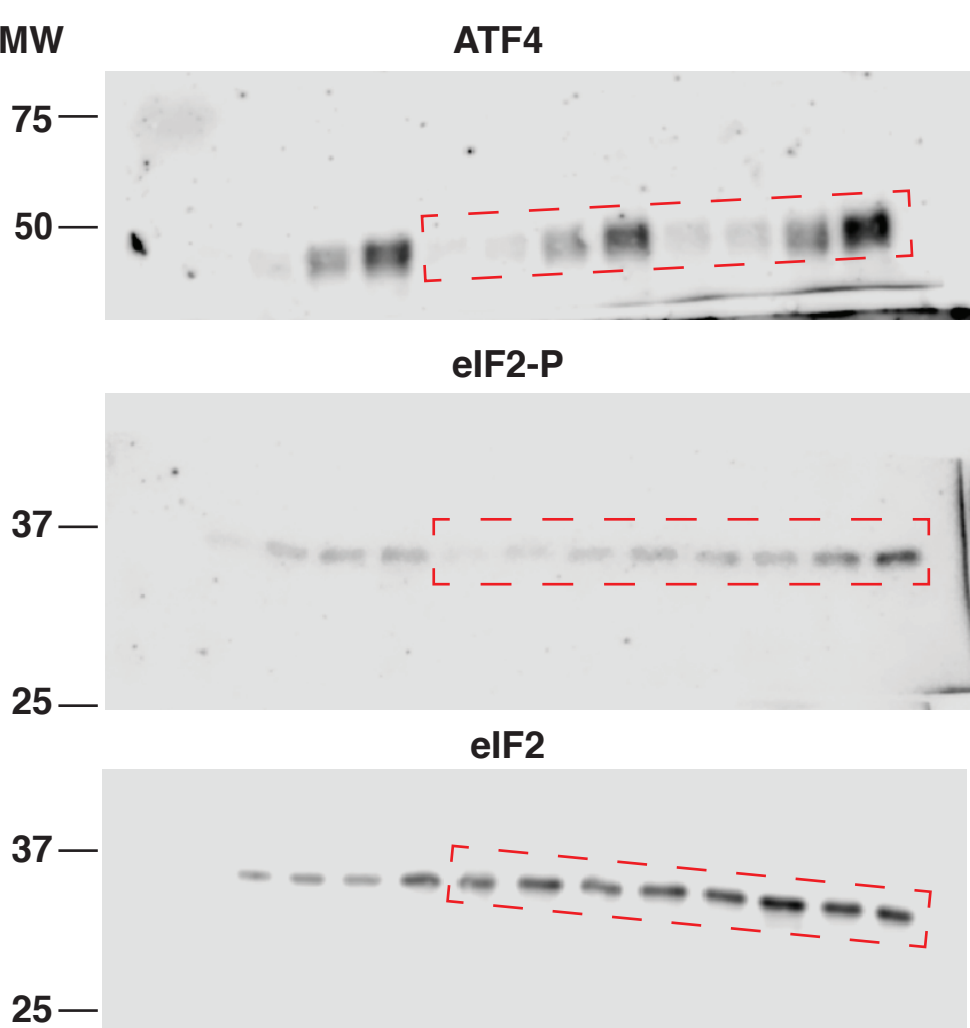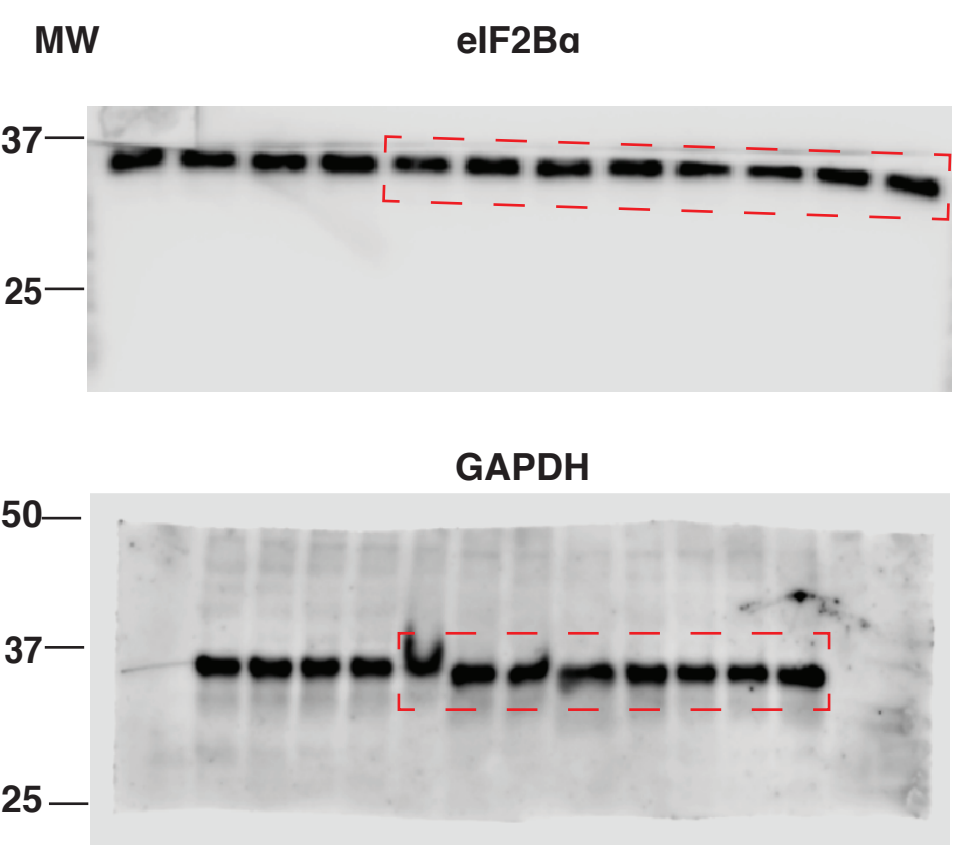

Supplement: Supplementary file 10 — Uncropped blots corresponding to Fig. 6. [file 41589_2023_1453_MOESM10_ESM.pdf]
